# The Red Queen model of recombination hot-spot evolution: a theoretical investigation

**DOI:** 10.1098/rstb.2016.0463

**Published:** 2017-11-06

**Authors:** Thibault Latrille, Laurent Duret, Nicolas Lartillot

**Affiliations:** Université Lyon 1, CNRS, UMR 5558, Laboratoire de Biométrie et Biologie Evolutive, Villeurbanne, France

**Keywords:** recombination, Red Queen, PRDM9, biased gene conversion

## Abstract

In humans and many other species, recombination events cluster in narrow and short-lived hot spots distributed across the genome, whose location is determined by the Zn-finger protein PRDM9. To explain these fast evolutionary dynamics, an intra-genomic Red Queen model has been proposed, based on the interplay between two antagonistic forces: biased gene conversion, mediated by double-strand breaks, resulting in hot-spot extinction, followed by positive selection favouring new PRDM9 alleles recognizing new sequence motifs. Thus far, however, this Red Queen model has not been formalized as a quantitative population-genetic model, fully accounting for the intricate interplay between biased gene conversion, mutation, selection, demography and genetic diversity at the PRDM9 locus. Here, we explore the population genetics of the Red Queen model of recombination. A Wright–Fisher simulator was implemented, allowing exploration of the behaviour of the model (mean equilibrium recombination rate, diversity at the PRDM9 locus or turnover rate) as a function of the parameters (effective population size, mutation and erosion rates). In a second step, analytical results based on self-consistent mean-field approximations were derived, reproducing the scaling relations observed in the simulations. Empirical fit of the model to current data from the mouse suggests both a high mutation rate at PRDM9 and strong biased gene conversion on its targets.

This article is part of the themed issue ‘Evolutionary causes and consequences of recombination rate variation in sexual organisms’.

## 1. Introduction

In mammals, as in many other species, meiotic recombination events are not uniformly distributed along chromosomes. Instead, they tend to occur within narrow regions, called hot spots, of a typical length of 1–2 kb [1–4]. Between 20 000 and 40 000 hot spots have been identified in humans [2,3], and over 40 000 in the mouse [4]. Strikingly, hot spots are not conserved between humans and chimps [5–7], nor between mouse subspecies [8], suggesting that recombination landscapes are highly dynamic.

At least in humans and the mouse, the location of hot spots is primarily determined by the Zn-finger protein PRDM9. Upon binding DNA at specific sequence motifs of approximately 10–20 bp through its Zn-finger domain [9–11], PRDM9 triggers the formation of a double-strand break (DSB) in the immediate vicinity of the target site [12]. This DSB is repaired, ultimately leading to a crossover (CO) or a non-crossover (NCO) recombination event. In both cases, the DNA sequence around (and including) the binding site of PRDM9 is resected from the chromosome and is repaired using the homologous chromosome as a template, over approximately 300–1000 base pairs—a process called gene conversion.

When the two chromosomes differ due to the presence of heterozygous sites, an event of gene conversion leads to a loss of information (loss of the allele carried by the broken chromosome). As a result, gene conversion has the following paradoxical consequence: if one of the chromosomes has a version of the binding site that partially or completely inactivates the binding of PRDM9 (cold allele), while the sequence on the other chromosome corresponds to a fully functional (hot) allele, then PRDM9 will preferentially target the chromosome carrying the hot allele—whose sequence will then be erased and converted into the cold version present on the homologue [13,14]. This process of DSB-induced biased gene conversion (biased in favour of cold alleles) will hereafter be referred to as dBGC. At the level of the population, dBGC results in a progressive accumulation of inactive PRDM9 target sites, by mutation and preferential transmission of the mutants, leading to an increase in frequency and the ultimate fixation of inactive hot spots genome-wide. Hence, through the action of dBGC, recombination hot spots turn out to be self-destructive, a phenomenon referred to as the hot-spot conversion paradox [15].

Population-genetic arguments suggest that dBGC might be a sufficient force to lead to the rapid extinction of recombination hot spots over the genome [14–16], thus raising the question of how recombination is maintained in the long run. A remarkable observation in this respect is that PRDM9 is the most rapidly evolving gene in primates and rodents, and more generally across metazoans [17–19], suggesting that the ZnFn domain of PRDM9 is subject to strong mutational and selective pressure, favouring new alleles targeting new sets of hot spots across the genome [20]. Thus, the interplay between dBGC, leading to the loss of current hot spots, and mutation and positive selection at the PRDM9 locus, leading to the recruitment of new hot spots, appear to provide a convincing model for explaining the rapid evolutionary turnover of recombination landscapes [10,21,22]. It is an instance of an intra-genomic conflict, where two processes are chasing after one another. As such, it is analogous to Red Queen situations, such as arms races between hosts and pathogens, often encountered in evolutionary ecology.

Several observations are consistent with the predictions of the Red Queen model of recombination turnover. First, the analysis of transmission segregation by sperm-typing at a few loci demonstrated that some recombination hot spots are subject to dBGC in humans and mice [13,23,24]. Second, comparative genome analyses have suggested that PRDM9 target motifs accumulate substitutions at an accelerated rate genome-wide [10,22,25,26]. Given that recombination is required for the proper segregation of chromosomes, and that PRDM9 knockout mice are sterile [27], it seems plausible that the loss of recombination hot spots might affect fertility, thus inducing positive selection on PRDM9 for eliciting new sequence motifs. Theoretical models indeed suggest that Red Queen dynamics could in principle explain the turnover of recombination landscapes in the context of dBGC [21].

In many respects, however, the Red Queen model is still speculative and needs to be further theoretically investigated and empirically tested. A first specific issue is how exactly positive selection is leveraged onto PRDM9 by hot-spot extinction. Second, a more accurate picture of the Red Queen model should really integrate the role of genetic diversity. Thus far, the Red Queen has been stated mostly in terms of a succession—global hot-spot extinction by dBGC followed by allelic replacement at the PRDM9 locus. However, in reality, the PRDM9 locus is known to be highly polymorphic, with many alternative alleles, each targeting a different subset of hot spots [17–19,25,26]. Whether the primary driver of such high levels of polymorphism is selection or mutation is not yet totally clear. The Zn-finger domain of PRDM9 has a minisatellite structure, and as a result, the entire domain follows a complex process of point mutation and concerted evolution by unequal meiotic CO. This appears to result in a particularly high mutation rate, up to 10^−5^ per generation [28], sufficient in itself to promote high levels of standing variation. The role of positive selection on PRDM9, on the other hand, and in particular, whether this selection is diversifying, is currently much less clear.

Whatever the primary cause of such high levels of polymorphism, the presence of multiple alleles at the PRDM9 locus in a population should distribute recombination rates more evenly across a larger number of weaker hot spots genome-wide, thereby weakening the effect of dBGC and thus slowing down extinction of old hot spots. Thus, there are potentially non-trivial feedbacks between standing diversity and other aspects of the stationary regime of the Red Queen process, in which demography is expected to play an important role. A question of particular interest in this context is how the stationary regime of the Red Queen (in terms of the mean levels of depletion of recombination rates, the equilibrium diversity at the PRDM9 locus and the rate of turnover of recombination landscapes) scales with population size and with the parameters of the genetic system (in particular, the mutation rates at the target sites and at the minisatellite locus encoding the Zn-finger domain of PRDM9).

Based on current empirical evidence, both dBGC and PRDM9 are likely to be implicated in the evolutionary dynamics of recombination landscapes across placental mammals, with the notable exception of the dog lineage [29]. On a broader scale, PRDM9 is present across the metazoan tree of life, although absent in birds [30] and in some other specific lineages. Across mammals, and even more so across metazoans, both mutation rates and effective population size are likely to vary over several orders of magnitude. Thus, if the Red Queen is to provide a model of the evolutionary dynamics of recombination, not just in humans and in the mouse, but more globally across mammals or metazoans, it is fundamental to better understand how the working regime of this model effectively scales as a function of its parameters.

With this in mind, here, we introduce a simple population-genetic model of the Red Queen of recombination turnover. The model was first implemented as a simulation program and run over a broad range of conditions, so as to explore its qualitative behaviour, as well as its scaling, as a function of the parameters. These simulation experiments were then backed up by analytical and numerical approximations, based on a self-consistent mean-field argument, which are meant to capture the main properties of the stationary regime of the process. Based on this analysis, we provide a general overview of the behaviour of the Red Queen model in different parameter regimes. Finally, an empirical calibration of the model against currently available data in the mouse was attempted, which suggests both a high mutation rate at PRDM9 and strong biased gene conversion on its targets.

## 2. Material and methods

### (a) Population-genetic model

The evolutionary dynamics of the Red Queen was formalized as a Wright–Fisher model with mutation and selection. The population is assumed to be panmictic, with constant size *N*_e_ and with non-overlapping generations. Only the genetic composition of the PRMD9 locus was explicitly modelled, the evolutionary dynamics at other loci (PRDM9 targets across the genome) being implicit.

#### (i) Mutation

The locus PRDM9 mutates at constant rate *u* per generation. Each mutation produces a new functional PRDM9 variant, endowed with an entirely new set of targets sites across the genome, all of which are assumed to recombine at the same rate. The number of targets is the same for each allele, and there is no overlap between the targets of distinct PRDM9 alleles. Here, we will not explicitly define the total number of targets nor the absolute recombination rate induced by each target, since the model turns out to be independent of those two quantities.

The rate *u* should be understood as a functional mutation rate (discounting loss-of-function mutants). At each generation, *K*_*t*_ denotes the number of PRDM9 alleles in the population, and ∀*i* ∈ {1, …, *K*_*t*_}, *n*_*i*,*t*_ is the number of copies of the *i*th allele in the population. Consequently, *x*_*i*,*t*_ = *n*_*i*,*t*_/2*N*_e_ is the frequency of allele *i* at time *t*. As usual, we define the *scaled mutation rate* *μ* = 4*N*_e_*u*.

#### (ii) Recombination and erosion of the targets by dBGC

The recombination activity induced by an allele is maximal at the birth of this allele. As time proceeds forward, however, the targets of the allele are progressively eroded by dBGC. This erosion is modelled implicitly, by tracking over time the fraction of active targets associated with each allele. This fraction is denoted as *θ*_*i*,*t*_ for allele *i* at time *t*. In the following, this fraction will be called the *activity* induced by allele *i*. Because of dBGC, this fraction is a decreasing function of time.

We assume an additive model for the genome-wide recombination induced by a given genotype. Specifically, consider a diploid individual, with alleles *i* and *j* at the PRDM9 locus. At time *t*, the activities of these two alleles are *θ*_*i*,*t*_ and *θ*_*j*,*t*_, respectively. The genome-wide recombination activity in this individual is then assumed to be proportional to

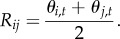
Note that this is assumed to be valid also for a homozygous individual (i.e. when *i* = *j*). Note also that *R*_*ij*_ is the relative recombination activity. The absolute recombination activity would be equal to *R*_*ij*_ multiplied by the total number of targets and the recombination rate induced by each target (which are not explicitly considered).

Mechanistically, the strength of dBGC depends on several factors: in particular, the rate at which a target site initiates DSB events and the probability that the site undergoes gene conversion upon initiating a DSB. Similarly, the recombination (i.e. CO) rate induced by a given hot spot depends on the rate of DSB initiation and on the probability that this initiation will result in a CO event, as opposed to a NCO or a resolution with the sister chromatid instead of the homologue. Here, we assume that all those factors are constant across all hot spots. Thus, in the end, we only need to consider the net rate *g*, per generation, at which a given mutant allele of the target site converts its wild-type homologue in an individual heterozygote at that target site and homozygous for the PRDM9 allele recognizing this target site.

Note that *g* is the conversion rate conditional on the PRDM9 genotype of the individual. On the other hand, at the level of the population, the conversion rate at this site will also depend on the rate at which DSB is initiated at that site, which in turn is proportional to the frequency of the PRDM9 allele in the population. Thus, for allele *i*, segregating at frequency *x*_*i*,*t*_ at time *t*, the conversion strength at a target site of this allele is equal to *gx*_*i*,*t*_.

As the mutation rate at the target sites *v* is typically low (*v* ∼ 10^−7^, thus 4*N*_e_*v* ≪ 1), at any given time, most targets associated with a given PRDM9 allele are either fully active, or fully inactive (i.e. a minor fraction of the targets are in a polymorphic state). As a result, the rate at which the activity induced by allele *i* over the entire genome decays is just the rate of substitution from active to inactive hot spots at the level of the population. This substitution rate is itself equal to the rate of inactivating mutations per target at the level of the population, 2*N*_e_*v*, multiplied by the fixation probability of the inactive mutant. Under strong dBGC, the fixation probability is equal to 2*gx*_*i*,*t*_. Altogether, the activity induced by allele *i* decays as follows:
2.1
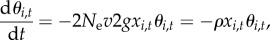
where we define the *scaled erosion rate* *ρ* = 4*N*_e_*vg*. Note that, under the mutation-fixation approximation considered here for the effect of dBGC on the targets, the behaviour of the Red Queen process depends on the mutation rate at the targets *v* and the strength of conversion *g* only through their product *vg*, which we call the erosion rate.

#### (iii) Selection

The fitness of an individual is assumed to be an increasing function *f* of its relative recombination activity *R* ∈ (0, 1). In the following, we will more specifically consider two alternative fitness functions:

— a power-law function:
2.2
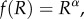
where *α* is a parameter of the model. Larger values of *α* induce a stronger selection against low recombination rates (electronic supplementary material, figure S1)
— an exponential function:
2.3
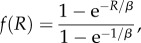
Here also, large values of *β* induce a stronger selection against low recombination rates (electronic supplementary material, figure S1).

The fitness functions are normalized so that *f*(1) = 1.

The fitness of an individual with genotype (*i*, *j*) is thus

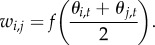
The average fitness induced by allele *i* over the population is then given by

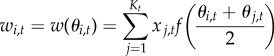
and the mean fitness over the population is

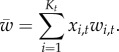
Finally, we define the selection coefficient *s*_*i*,*t*_ associated with allele *i* at time *t* as

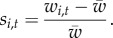
In particular, the selection coefficient associated with a new allele entering the population, which will be denoted by *s*_0_, is
2.4
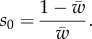


#### (iv) Overall simulation cycle

For each new generation, the simulation is decomposed in three steps, performed in the following order:

— Mutation and creation of new alleles of PRDM9: the number of new alleles is drawn from a Poisson distribution with mean 2*N*_e_*u*. Each new allele replaces a randomly chosen resident allele;
— Erosion: the activity of each allele currently segregating in the population is eroded by a factor proportional to the frequency of the allele: 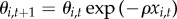, for *i* = 1..*K*_*t*_;
— Drift and selection: the new generation of 2*N*_e_ haploid copies is drawn from a multinomial distribution. The probability of drawing allele *i* is equal to its frequency *x*_*i*,*t*_ multiplied by its relative fitness 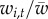.

The model was implemented in Python, the code is hosted athttps://github.com/ThibaultLatrille/RedQueen. Each simulation starts by a burn-in, and the data are recorded only once the steady state is reached. The burn-in phase is finished when all initial alleles in the population are extinct and replaced by new alleles. The number of generations simulated at the steady state is 50 times the number of generations that were needed to achieve the burn-in.

#### (v) Model with variation in strength across hot spots

The model introduced thus far assumes that all active hot spots associated with a given PRDM9 allele recombine at the same rate. A second model was also considered, allowing for variation in the recombination rate across hot spots, according to a gamma distribution of mean 1 (because we consider only relative recombination rates) and shape parameter *a*:

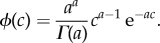
Under this model, the relation between the distribution of hot-spot activity and the mean recombination rate over the genome induced by a given allele is more complex than under the uniform-rate model considered above. Specifically, for a given relative recombination rate *c* > 0, we define *θ*_*c*,*i*,*t*_ as the fraction of those hot spots recombining at relative rate *c* that are still active at time *t* for allele *i*. Then, the overall fraction of active targets for allele *i* at time *t* is given by

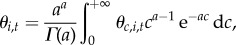
while the mean relative recombination rate induced by allele *i* over the genome at time *t* is

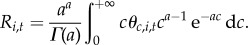
In addition, as we assume that conversion strength and recombination rates are proportional, the rate of erosion for the fraction of hot spots recombining at rate *c* decays at a rate proportional to *c*, i.e.

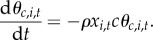


### (b) Summary of the model parameters

Altogether, the model effectively depends on four parameters: the effective population size *N*_e_, the erosion rate *vg*, the mutation rate *u* at the PRDM9 locus and the parameter of the fitness function (or fitness parameter), which is either *α* (under the power-law fitness function) or *β* (when using the exponential fitness function). The model allowing for variation in the recombination rate across hot spots also depends on the shape parameter *a*, thus giving a total of five parameters for this model.

In the following, three compound parameters will turn out to be of particular importance. First, the scaled erosion rate *ρ* = 4*N*_e_*vg* and the scaled mutation rate at the PRDM9 locus *μ* = 4*N*_e_*u*, which were already introduced above. Second, the compound parameter *ε* = *ρ*/*μ* = *vg*/*u*. This parameter measures the relative strength of the two arms of the Red Queen (erosion, at rate *ρ*, versus restoration of recombination, at rate *μ*), and thus determines the *erosion–restoration balance* of the Red Queen. Intuitively, for small *ε*, the mutational input at the PRDM9 locus dominates over the rate of erosion, and thus the equilibrium level of erosion of recombination landscapes is expected to be low. Conversely, for large *ε*, the stationary regime of the Red Queen is expected to be characterized by high erosion levels. Note that *ε* does not depend on *N*_e_.

### (c) Summary statistics

To explore the behaviour of the model as a function of the parameters, several summary statistics were considered. These statistics are meant to capture key features of the Red Queen dynamics: the diversity at the PRDM9 locus, the mean recombination rate over the population at stationarity and the time of turnover of the genetic diversity at the PRDM9 locus (or, equivalently, the time of turnover of recombination landscapes). These summary statistics are computed at stationarity and are averaged over long simulation trajectories.

#### (i) Diversity of PRDM9

Diversity can be defined in several ways; for example, one could consider the number of distinct alleles *K*_*t*_. One shortcoming of this definition is that *K*_*t*_ is strongly sensitive to the behaviour of low-frequency PRDM9 alleles, most of which never invade the population and therefore do not meaningfully contribute to the macroscopic behaviour of the model. An alternative, more relevant, measure of diversity is the inverse of the homozygosity:

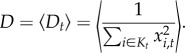
Note that *D* equals *K* if all alleles have equal weights and is close to 1 if one allele dominates the population. Thus, *D* can be seen as the *effective* number of PRDM9 alleles.

#### (ii) Mean relative recombination rate

As the model linking the activity of each allele (the fraction of active targets) with genome-wide recombination is additive, the average relative genome-wide recombination rate at the population level is equal to

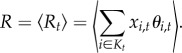


#### (iii) Turnover time

The turnover time is defined as the decorrelation time of the diversity at the PRDM9 locus. This can be quantitatively assessed using the *cross-homozygosity* (CH), which is defined as the fraction of homozygotes in a population that would be obtained by hybridizing populations at time *t* and *t* + *T* in equal proportions. The CH reduces to the regular (or instant) homozygosity for *T* = 0 and drops to 0 for large *T*. The turnover time is defined as the time *T* for which the cross-heterozygosity is equal to half of the instant homozygosity.

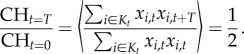


A series of phase diagrams, showing the scaling relations between summary statistics and parameters of the model, were obtained as follows. For each parameter, 64 independent simulations were run, each time changing the value of only one parameter around its central value. The range around the central value of the parameter is 10^4^. The mean and the variance of each summary statistic was calculated for each simulation, and plotted as a function of the parameter.

## 3. Results

### (a) Simulation results

**Figure 1.**
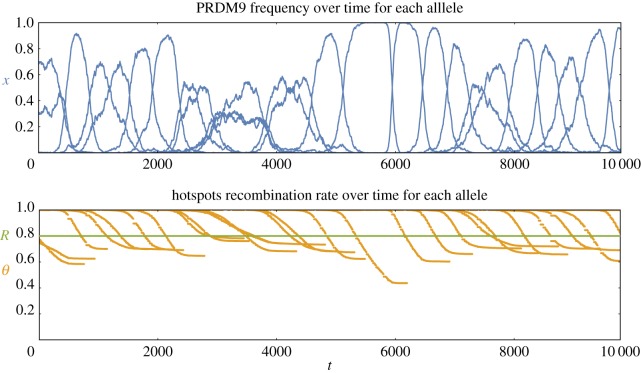
A typical simulation trajectory of the Red Queen model, with allele frequencies (*x*, top) and allele activity (*θ*, bottom, orange) through time. The green line represents the mean recombination activity (mean of *θ*) over the simulation. (Online version in colour.)

A typical simulation trajectory is displayed in figure 1. As time proceeds forward, new PRDM9 alleles, created by mutation, invade the population and increase in frequency, reaching a peak and then decreasing until being lost and replaced by new alleles figure 1*a*. Tracking the activity of each allele through time (figure 1*b*) shows that new alleles start with maximal recombination activity. Progressively, however, their targets are eroded by dBGC. When the activity induced by an allele goes below the average activity over the population (whose equilibrium value over time, *R*, is shown as a straight line in figure 1*b*), this allele starts to be selected against, decreasing in frequency, until being eliminated from the population. Of note, the activity of a dying allele is typically not 0. Instead, it reaches a finite level, whose average value (*R*_∞_) will be considered further below.

**Figure 2.**
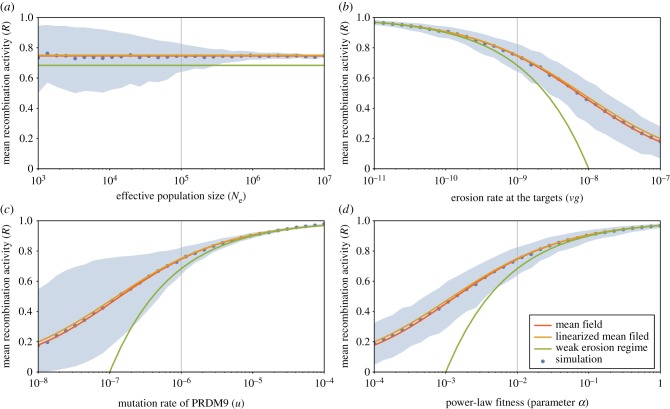
Mean recombination activity *R*, as a function of effective population size *N*_e_ (*a*), erosion rate *vg* (*b*), mutation rate at the PRDM9 locus *u* (*c*) and the fitness parameter *α* (*d*) under the power-law fitness model. Mean-field approximations, either linearized (orange) or generalized (red) and weak erosion approximation (green) are shown on the top of the mean and variance over the simulations (blue). For plots under the exponential fitness model (see electronic supplementary material, figure S3). (Online version in colour.)

**Figure 3.**
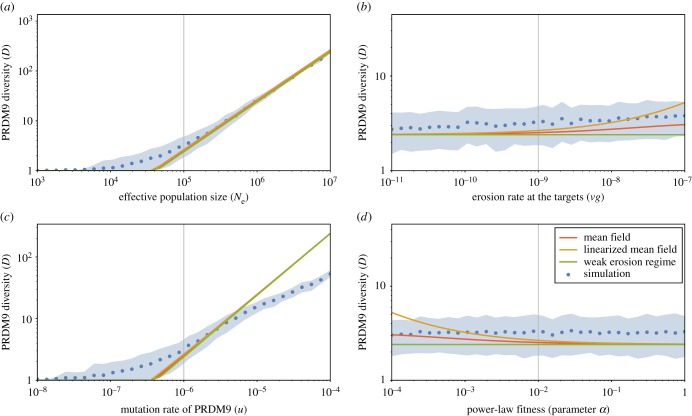
Genetic diversity at the PRDM9 locus *D*, as a function of effective population size *N*_e_ (*a*), erosion rate *vg* (*b*), mutation rate at the PRDM9 locus *u* (*c*) and the fitness parameter *α* (*d*) under the power-law fitness model. Mean-field approximations, either linearized (orange) or generalized (red) and weak erosion approximation (green) are shown on the top of the mean and variance over the simulations (blue). For plots under the exponential fitness model (see electronic supplementary material, figure S4). (Online version in colour.)

**Figure 4.**
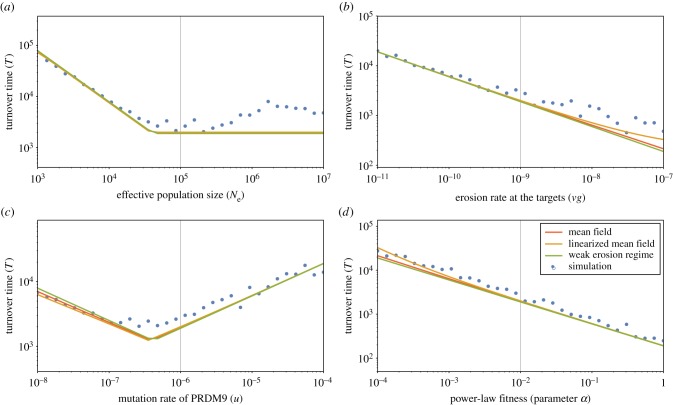
Turnover time *T*, as a function of effective population size *N*_e_ (*a*), erosion rate *vg* (*b*), mutation rate at the PRDM9 locus *u* (*c*) and the fitness parameter *α* (*d*) under the power-law fitness model. Mean-field approximations, either linearized (orange) or generalized (red) and weak erosion approximation (green) are shown on the top of the mean and variance over the simulations (blue). For plots under the exponential fitness model (see electronic supplementary material, figure S5). (Online version in colour.)

As a way to capture the key features of the Red Queen dynamics at stationarity, three summary statistics were considered: mean recombination activity, genetic diversity at the PRDM9 locus and turnover time (i.e. the decorrelation time of genetic composition at the PRDM9 locus). For a given configuration of parameters of the model (effective population size *N*_e_, mutation rate at the PRDM9 locus *u*, erosion rate at the targets *vg*, fitness parameter *α* or *β*), these three statistics were averaged over long simulation trajectories. Their scaling behaviour as a function of the four main parameters are displayed on figures 2–4. Several points are noteworthy.

First, the equilibrium recombination rate is suboptimal: *R* < 1 (figure 2). Equivalently, there is a recombination *gap* 1– *R* caused by the Red Queen. One question of interest, which will be further considered below, is to characterize and quantify this recombination gap. Another important observation is that the equilibrium recombination rate (*R*) is constant as a function of *N*_e_. It increases with *u* and *α* and decreases with *vg*.

Second, plotting PRDM9 diversity (*D*) as a function of *N*_e_ and *u* (figure 3) reveals two distinct regimes. The transition between the two regimes appears to essentially depend on the scaled mutation rate (*μ* = 4*N*_e_*u*) at the PRDM9 locus. For low scaled mutation rates (*N*_e_*u* ≪ 1), at any given time, a single PRDM9 allele dominates the population (*D* = 1). In this *succession* regime, PRDM9 alleles replace each other through hard sweeps—which corresponds to the typical verbal description of the Red Queen dynamics. In this regime, increasing *N*_e_ merely accelerates the dynamics (turnover time, figure 4). At any time, recombination is concentrated on one single set of recombination hot spots (corresponding to the currently dominating PRDM9 allele), and thus the variance of recombination landscapes is maximal.

On the other hand, when *N*_e_*u* is sufficiently high, successive waves of erosion–invasion start to overlap, such that multiple PRDM9 alleles now coexist in the population. In this polymorphic regime, diversity at the PRDM9 locus is roughly proportional to *N*_e_*u* (figure 3) and does not strongly depend on the erosion rate *vg* nor on the fitness parameter *α*. Furthermore, the consequence of an increase in *N*_e_ is not anymore an acceleration of the dynamics, as in the succession regime. Instead, when *N*_e_ increases, PRDM9 diversity increases, such that recombination spreads over a larger number of more weakly recombining hot spots, thus resulting in an overall decrease in the variance of recombination landscapes at the level of the population. Conversely, the fact that the recombination hot spots become weaker exactly compensates for the increase in the strength of biased-gene conversion caused by the larger effective population size, and thus hot-spot lifetime (turnover time) now remains constant as a function of *N*_e_ (figure 4).

### (b) Analytical approximations: a linearized mean-field argument

In the following, we derive analytical approximations of the Red Queen model introduced above. These approximations are meant to capture the scaling relations between summary statistics and model parameters observed in the simulation experiments. They will also provide mechanistic insights into the inner working of the Red Queen process.

The two regimes, succession and polymorphic, are considered in turn. In both cases, the general structure of the argument is as follows. In principle, the changes in frequency of a typical PRDM9 allele are determined by a combination of selective effects and random drift. Furthermore, selection normally depends on the relative difference between the fitness of the allele and the constantly fluctuating fitness background of the population. Our main simplifications are to (i) ignore stochastic effects and to consider a deterministic version of the model (strong selection); (ii) to consider that the mean fitness of the population is constant through time, being equal to its time average (mean-field approximation); (iii) to assume that the differences in fitness between alleles are small, so that we can linearize the fitness function as a function of the *θ*_*i*_'s (linear model). A more general version of the model not making assumption (iii) will be considered in the next subsection.

Linearizing the model allows us to express the mean fitness of the population, and therefore also the selection coefficient experienced by a new allele entering the population, as a function of *R*, the mean recombination activity in the population. This selection coefficient determines the mean waiting time *τ* between successive invasions of the population by new alleles. Thus, we can express *τ* as a function of *R* and the parameters of the model. Conversely, knowing the waiting time before the next invasion, and accounting for ongoing erosion of recombination for resident alleles over this time leads to an estimate of the mean equilibrium recombination activity of the population *R*, as a function of *τ*. Combining these two relations and eliminating *τ* leads to a self-consistent mean-field estimate of *R*. From there, we can derive estimates for all other summary statistics.

The self-consistent estimate of *R* obtained using this approach is implicit: it is the solution of an equation of the form *R* = *g*(*R*), which can be solved only numerically. An explicit approximate solution will be derived under the additional assumption that the recombination gap is small 1 − *R* ≪ 1 (weak erosion approximation).

#### (i) Analytical approximations in the succession regime

In the succession regime, there is essentially one PRDM9 allele at a time, whose frequency is close to 1. As a consequence, and from equation (2.1), the recombination activity associated with this allele decreases at constant rate *ρ* = 4*N*_e_*vg*:

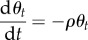
and thus *θ*_*t*_ follows an exponential decrease through time:

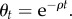
Let us call *τ* the mean time between two successive invasions. We now derive two independent relations between *τ* and *R*.

First, we can derive an approximation for the rate of invasion of the population by a new PRDM9 allele using a simple population genetic argument. This rate is equal to the rate of mutation at the PRDM9 locus at the level of the population, 2*N*_e_*u*, multiplied by the probability of invasion, which is itself equal to 2*s*_0_, where *s*_0_ is the selection coefficient experienced by the new allele (equation (2.4)). This coefficient depends on the current activity of the resident allele, *θ*_*t*_ (the activity of the invading allele is 1). After linearization, it can be expressed as

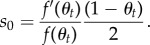
In principle, this selection coefficient depends on time, through *θ*_*t*_. However, we can approximate it by a constant coefficient, by just replacing *θ*_*t*_ by the average activity *R* in this equation:

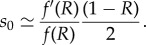
This leads to the following estimate for the rate of invasion:

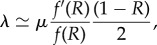
where *μ* = 4*N*_e_*u* is the scaled mutation rate at the PRDM9 locus. The inverse of *λ* gives an approximation to *τ*, thus leading to our first relation:
3.1
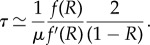


Second, the mean recombination activity is simply the time average of *θ*_*t*_ over the time where the allele dominates the population. This time is (on average) equal to *τ*, thus leading to the following approximation:
3.2
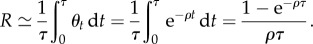
Noting that the minimum activity of a dying allele, at the time where it is being replaced by a new allele (i.e. at time *τ*), is equal to
3.3
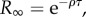
equation (3.2) can be rewritten as:

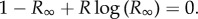
This relation between *R* and *R*_∞_ will also be found in the polymorphic regime.

Finally, substituting *τ* in equation (3.2) by its expression given by equation (3.1) leads to a self-consistent equation on *R*, which, after some algebra, can be expressed in compact form as
3.4
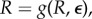
where *g* is a numerical function and *ε* = *vg*/*u* is the erosion–restoration balance parameter (see summary of model parameters in the Material and methods section). This self-consistent relation has a unique solution (electronic supplementary material, figure S2, in the case of a power-law fitness function), which can be obtained numerically, giving an estimate for *R*. Note that this self-consistent solution for *R* implies that *R* depends on the parameters of the model only through *ε*, which is itself independent of *N*_e_. As a result, the solution *R* is also independent of the effective population size, as our simulation was suggesting.

Once a numerical estimate of *R* is available, all other quantities of interest can be expressed as a function of *R* and of the parameters of the model. The diversity at the PRDM9 locus *D* is simply equal to 1 because there is only one allele. As for the turnover time *T*, it is simply equal to the mean time between two successive invasions *τ*, already given above as a function of *R* (equation (3.1)). Note that this time is inversely proportional to *N*_e_. In other words, in the succession regime, changes in *N*_e_ merely change the timescale of the Red Queen dynamics, without any effect on the stationary state.

#### (ii) Analytical approximations in polymorphic regime

Ignoring random drift and linearizing the fitness as a function of the activities, a closed set of differential equations for the frequencies of PRDM9 alleles (*x*_*i*,*t*_) and their associated recombination rate (*θ*_*i*,*t*_) can be derived:
3.5
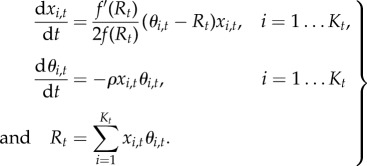


Under the assumption of many alleles co-segregating in the population, *R*_*t*_ = *R* is essentially constant, owing to an averaging effect over the specific trajectories of each allele. This leads to a decoupling of the above system of equations. As a result, we can focus on the trajectory of a single typical allele, with frequency *x*_*t*_ and relative recombination rate *θ*_*t*_:
3.6
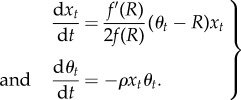
Here, *R* is now an external parameter, which will be determined in a second step, using a self-consistent argument.

**Figure 5.**
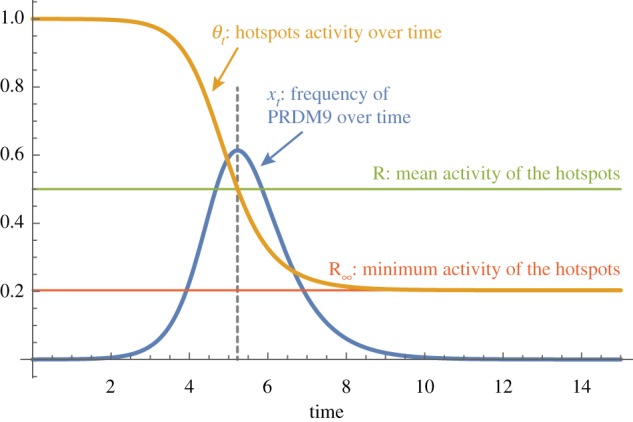
Trajectory of the frequency and the activity of a typical allele through time, under the deterministic and mean-field approximation (see text for details). (Online version in colour.)

A numerical solution of this system of equations, for a given value of *R*, is shown in figure 5: starting from a low frequency and a maximal activity, a typical allele first increases in frequency. Concomitantly, its activity is progressively eroded, at an increasingly higher rate. When the activity reaches the (now externally given) *R*, the allele frequency reaches its maximum, after which it starts to decrease, ultimately going to 0. Meanwhile, the activity converges to a strictly positive asymptotic value *R*_∞_.

This system of equations does not have an analytical solution as a function of *t*. On the other hand, *θ*_*t*_ is monotonic, and *x* can be analytically expressed as a function of *θ*_*t*_:

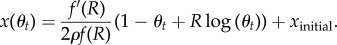


Letting *t* → ∞, *θ*_*t*_ converges to *R*_∞_ and *x*(*θ*_*t*_) to 0. In addition, as *x*_initial_ = 1/*N*_e_ is small, we can let it go to 0, which yields a relation between *R* and *R*_∞_:
3.7
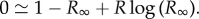
Note that this is the same equation as in the succession regime.

**Figure 6.**
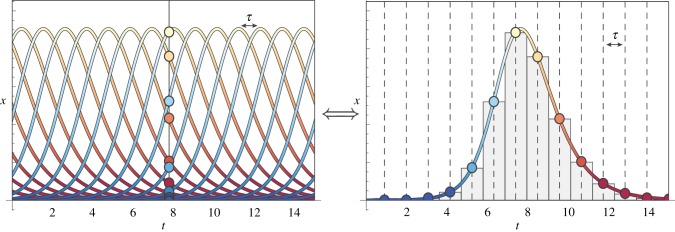
A tiling principle. On the left, An idealized realization of the Red Queen dynamics is depicted, in which new alleles invade the population at regular time intervals, all equal to *τ*, and have the same allele frequency trajectory. Taking the sum of allele frequencies at any given time (vertical bar), which by definition is equal to 1, is then equivalent to summing the values taken by the frequency of one specific allele at regular time intervals (right). Multiplying this sum by *τ* amounts to approximating the integral 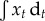 by the histogram shown on the right panel. Thus, 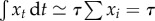. This argument can more generally be used to approximate any sum of the form 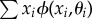 by the corresponding integral 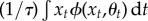. (Online version in colour.)

So far, we have considered *R* as an external parameter. Now, if all alleles have the same trajectory but different arrival times, they all contribute to *R*. Using this argument, we can get a self-consistent estimation for *R*. This can be done by deriving two independent relations between *τ* and *R*. First, using a tiling principle (figure 6), we note that
3.8
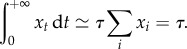
Although *x*_*t*_ is not analytically available as a function of *t*, the integral can be analytically computed (electronic supplementary material, appendix S1), leading to the following relation:

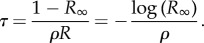
Inverting this equation gives

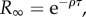
which is the same expression for *R*_∞_ as that found in the succession regime (equation (3.3)). Replacing *R*_∞_ by this expression in equation (3.7) then gives
3.9
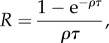
again, as in the succession regime (equation (3.2)).

Second, relying on the population-genetic argument used in the context of the succession regime, we can identify the time between two successive invasions *τ* as the inverse of the rate of invasion *λ*, which is equal to the mutation rate multipled by 2*s*_0_, where *s*_0_ is the selection coefficient experienced by the newly invading allele, in a background recombination activity equal to *R*:
3.10
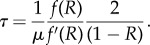
Although derived using different arguments, these two relations, equations (3.9) and (3.10), are the same as those obtained under the succession regime (equations (3.2) and (3.1)). Combining them together therefore leads to the same self-consistent equation for *R* (equation (3.4)), which gives us a numerical estimate of *R* also valid in the polymorphic regime.

Once a numerical estimate of *R* is available, other summary statistics can be computed as a function of *R*. Thus, using the same tiling argument as above, the diversity at the PRDM9 locus *D* can be obtained from

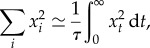
which is also analytically available (electronic supplementary material, appendix S1). Finally, the turnover time *T* can be determined by observing that, if *D* is to be interpreted as the effective number of alleles at the PRDM9 locus, then the genetic diversity at this locus will be entirely renewed after *D* successive invasions, each of which takes on average a time *τ*. Therefore, *T* = *Dτ*, with *D* and *τ* given above as a function of *R*, *R*_∞_ and the parameters of the model.

### (c) A more general derivation of the mean-field argument

The derivation conducted in the last section relies on a linearization of the fitness function, which allows us to express all quantities of interest as functions of the mean recombination activity *R*, and thus express the self-consistent mean-field argument directly on *R*. However, this linear approximation is not well suited to all fitness models. An alternative, more general, derivation of the self-consistent mean-field argument can be conducted, which can be used for arbitrary fitness schemes. This derivation can also be generalized to more complex models, in particular, allowing for variation in recombination rates across hot spots (next section).

The whole derivation is given in electronic supplementary material, appendix S2 under the polymorphic regime. Briefly, the idea is to first generalize the tiling argument introduced in the last section, which essentially expresses that, at stationarity, averaging over the population at any given time is equivalent to averaging over the trajectory of a typical allele. Using this argument, we can express the selection coefficient *s*_0_ associated with a newborn allele as an integral depending on the distribution of activities in the population. This integral is a function of the compound parameter *τ*. Conversely, given *s*_0_, and using the same argument as in the last section, we can derive an estimate of *τ*, as the inverse of *λ* = 4*N*_e_*us*_0_. Combining these two equations yields a self-consistent relation, now on the selection coefficient *s*_0_. The solution to this equation can be found numerically and can then be used to compute all summary statistics of interest.

### (d) Allowing for variation among hot spots in recombination rate

Thus far, the model assumes that all active hot spots have the same recombination rate (and thus are subjected to the same conversion strength). In reality, there is a substantial variance across hot spots in their recombination rate [3,4]. In this section, we explore a generalization of the Red Queen model that relaxes this uniformity assumption, by proposing that the distribution of relative recombination rates across the hot spots associated with a newborn PRDM9 allele is a gamma distribution of mean 1 and shape parameter *a* (with smaller value of *a* inducing higher variance across hot spots). On the other hand, all newborn PRDM9 alleles are still assumed to induce the same distribution of recombination activity over the genome (and, in particular, the same total recombination activity).

This new version of the model was explored only under the mean-field approximation, not backed up by explicit simulations. The mean-field derivation is essentially the same as the generalized version introduced in the previous section, with the only difference that the integral equation for *s*_0_ is now computed by averaging over the gamma distribution for a given allele of a given age. This again leads to a self-consistent relation on *s*_0_ which can be solved numerically (see electronic supplementary material, appendix S3 for details).

As before, once the self-consistent solution is found, the summary statistics (in particular, the mean recombination activity *R*) at the equilibrium set point can be computed. In addition to *R*, the mean fraction of active targets per allele, which we call *H*, is another statistic of interest, for which direct empirical evidence is available [25,26]. Thus far, without variance across hot spots in their basal recombination rate, the two statistics *R* and *H* were identical. However, this is not anymore the case under the gamma model now considered. Both *R* and *H* can be analytically computed as a function of *ρτ* (electronic supplementary material, appendix S3).

### (e) General scaling behaviour

#### (i) Scaling relations as a function of *N*_e_

Even without any explicit or any numerical solution for *s*_0_, the relations derived above for the recombination activity *R*, the mean time between successive invasions *τ*, the selection coefficient *s*_0_ acting on new PRDM9 alleles, the turnover time *T* and the diversity *D* at the PRDM9 locus imply the following scaling relations for all of these quantities as a function of *N*_e_. First, *s*_0_ is the solution of a self-consistent equation that depends only on the erosion–restoration balance parameter *ε* = *vg*/*u* (electronic supplementary material, appendix S2) and therefore does not depend on *N*_e_. The same thing is true more generally for *ρτ* and thus also for all quantities that depend only on *ρτ*: in particular *R* and *T*. As *ρ* = 4*N*_e_*vg* itself is directly proportional to *N*_e_ and as *ρτ* does not depend on *N*_e_, *τ* is inversely proportional to *N*_e_. In turn, as *T* = *τD* is constant as a function of *N*_e_, *D* is proportional to *N*_e_.

All of those relations are valid in the polymorphic regime. In the succession regime, the main difference is that *D* = 1, by definition. Otherwise, *R*, *τ* and *s* are still constant as a function of *N*_e_, and *T* is now inversely proportional to *N*_e_.

#### (ii) First-order development in the weak erosion regime (*ε* ≪ 1)

Throughout the self-consistent derivation presented in the last sections, the compound parameter *ε* = *vg*/*u* plays a key role. This parameter captures the relative strength of the two antagonistic forces of the Red Queen process, the rate of erosion of PRDM9 targets by dBGC, proportional to *vg*, and the rate of invasion by new PRDM9 variants, proportional to *u*. The equilibrium set point (or erosion–restoration balance) of the Red Queen process, in terms of the realized value of *R*, is essentially detemined by the relative magnitude of these two forces. Thus, if *ε* ≫ 1, erosion is strong compared to elicitation of new PRDM9 alleles, and *R* is expected to be small. Conversely, if *ε* ≪ 1, *R* is close to 1.

The weak erosion case (*ε* ≪ 1) lends itself to a simple first-order approximation of the self-consistent mean-field solution. This approximation may not be strictly valid in the empirically relevant regimes where depletion levels seem to be substantial (see below). Nevertheless, it leads to simple expressions for all summary statistics of interest in the stationary regime, thus giving a very concise (if somewhat idealized) summary of the scaling of the stationary regime of the Red Queen as a function of the parameters of the model.

The details of this first-order development are given in electronic supplementary material, appendix S4. In the end, all of the scaling relations can be expressed in terms of two compound parameters, *ε* = *vg*/*u* and *N*_e_*u*. In this regime, the scaling relations are then as follows: in the polymorphic regime, *ρτ*, *s* and 1 − *R* scale as 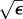, *T* as 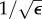, and *D* as *N*_e_*u*. In the succession regime, the only difference is that *D* = 1 and that *T* now scales as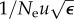.

## 4. Comparing analytical approximations with simulation results

The scaling relations predicted by our mean-field approximations, both linearized (green) and generalized (red), as well as the weak erosion approximation (orange), are shown on the top of the simulation results (under the power-law fitness model, with central parameter values *α* = 0.01, *u* = 10^−6^, *vg* = 10^−9^ and *N*_e_ = 10^5^), for the mean equilibrium recombination level *R* (figure 2), the genetic diversity *D* (figure 3) and the turnover time *T* (figure 4). In spite of the rather bold approximations that were made, the predictions of both the linearized and the generalized mean-field solutions agree very well with the simulation results. Expectedly, the weak erosion approximation fails when the recombination gap becomes significant (typically, when 1 − *R* is larger than 0.2).

One specific but notable deviation of the mean-field approximations from the simulation results occurs for the predicted equilibrium diversity *D* in the regime of a high scaled mutation rate, *N*_e_*u* ≫ 1 (electronic supplementary material, figur S3). A plausible explanation for this discrepancy is that we did not account, in our mean-field development, for the decrease in the frequency of a typical PRDM9 allele between successive generations directly due to the mutation pressure. This approximation is expected to be invalid whenever *s* and *u* are of the same order of magnitude.

The linearized version of the mean-field solution is less accurate under the exponential fitness model and for small values of the fitness parameter *β* (electronic supplementary material, figures S3–S6), probably because the exponential fitness function shows a stronger concavity at the equilibrium set point. In this regime, on the other hand, the generalized mean-field solution is accurate. Of note, under the exponential fitness function, the weak erosion approximation tends to break down for smaller recombination gaps than under the power-law fitness function.

Finally, a key assumption of the mean-field derivation is that selection on PRDM9 is sufficiently strong so that we can ignore random drift. In the parameter regimes considered thus far, this turns out to be the case: the scaled selection coefficient 4*N*_e_*s*_0_ at stationarity is always greater than 10, and usually of the order of 100 (electronic supplementary material, figure S6 under the exponential fitness function). However, for other parameter regimes (in particular, a smaller *N*_e_ or a smaller value for the fitness parameter *α* under the power-law fitness model), selection is not so strong compared to random drift (with a scaled selection coefficient 4*N*_e_*s*_0_ estimated under the mean-field approximation to be below 10). In this weaker selection regime, both versions of the mean-field approximation start to be substantially less accurate (electronic supplementary material, figures S7 and S8).

In summary, our mean-field derivation, at least in its generalized version, is accurate under all strong-selection regimes (in practice, whenever the self-consistent estimate of the scaled selection coefficient 4*N*_e_*s*_0_ > 10). Its linearized form is accurate as long as the log-fitness function is not too concave. And finally, the first-order weak erosion regime approximation (*ε* ≪ 1) is, rather expectedly, valid only for small equilibrium recombination gaps (1 − *R* < 0.2).

## 5. Scaling of the model allowing for variation in hot-spot strength

The model allowing for variation in hot-spot strength was explored under the generalized mean-field approximation, for several values of the shape parameter, ranging from low (*a* = 5) to high (*a* = 0.5) variance across hot spots (electronic supplementary material, figures S9–S12). In the limit *a* → ∞, this model reduces to the uniform model considered thus far, which is also indicated on the figures for comparison.

Under a fixed parameter regime, the mean fraction of active targets at equilibrium *H* (electronic supplementary material, figure S9) tends to increase as the variance increases (i.e. as the shape parameter *a* decreases). By contrast, the equilbirium recombination rate *R* (not shown) tends to decrease with decreasing *a*. This opposite behaviour of *R* and *H*, when *a* varies, is due to the fact that strongest hot spots are the first to be eroded, and thus, the depletion of the genome-wide recombination activity at equilibrium is mostly contributed by the extinction of a relatively minor fraction of very strong hot spots. This effect is stronger for smaller values of the shape parameter *a*.

The equilibrium diversity also tends to decrease with increasing variance (decreasing *a*) under most parameter regimes (electronic supplementary material, figure S10), except for extremely high conversion rates *vg* or extremely weak selection regimes (very small *α*). Correlatively, the turnover time (which is directly proportional to diversity), also tends to decrease with increasing variance across hot spots (not shown). Globally, however, the equilibrium regime of the Red Queen process is only moderately dependent on the variation in strength across hot spots (on the exact value of *a*).

### (a) Empirical calibration

The model can be tentatively calibrated against current empirical evidence in the mouse as follows. First, some of the parameters of the model can be fixed *a priori*. The effective population size in mouse subspecies is of the order of 10^5^ [19]. Assuming a point mutation rate of 10^−8^ per generation and an effective number of 10 inactivating mutations per target, *v* can be estimated to be of the order of 10^−7^. DBS initiation maps inferred by Chip-Seq [8] suggest that the distribution of DSB initiation is approximately exponential (i.e. gamma with a shape parameter *a* = 1). This leaves us with three parameters to estimate, *u*, *g* and the fitness parameter *α* under the power-law model.

These three parameters can be estimated by constraining the model using the three summary statistics *D*, *S*_0_ and *H*. In each of the three mouse subspecies, *castaneus*, *domesticus* and *musculus*, there is one major PRDM9 allele segregating at a frequency of the order of 30%, with most other alleles being much rarer [18,19], thus suggesting a value of *D* between 5 and 10 in each subpopulation. Experiments on hybrids between subspecies show that the major allele of each subspecies has eroded a fraction of its targets of the order of *H* ≃ 50% [25,26]. Finally, patterns of non-synonymous versus synonymous variation clearly indicate the presence of strong positive selection acting on the Zn-finger array of PRDM9 [17,19], suggesting a scaled selection coefficient *S*_0_ well above 1.

Altogether, this defines empirically reasonable values for the three summary statistics (*D* = 10, *H* = 0.5 and *S*_0_ = 10), which could be qualitatively reproduced by manual adjustment of the three free parameters of the model, giving the following estimates of *α* = 10^−4^, *u* = 3 × 10^−6^ and *g* = 3 × 10^−3^ (table 1, first row)—thus suggesting a high mutation rate *u* and a strong dBGC.

**Table 1.** Summary statistics predicted by the model with variation in hot-spot strength, under the generalized mean-field solution, for different combination of parameter values (in all cases, *a* = 1, *v* = 10^−7^). *H*: mean fraction of active targets per allele, *D*: PRDM9 diversity, *S*_0_ = 4*N*_e_*s*_0_: scaled selection coefficient associated with a new PRDM9 allele entering the population, *T*: turnover time.

*u*	*g*	*α*	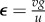	*H*	*D*	*S*_0_	*T*
3 × 10^−6^	3 × 10^−3^	1 × 10^−4^	1 × 10^−4^	0.6	9.9	26	6.4 × 10^4^
3 × 10^−6^	3 × 10^−4^	1 × 10^−4^	1 × 10^−5^	0.82	8.2	8.6	1.6 × 10^5^
3 × 10^−7^	3 × 10^−4^	1 × 10^−4^	1 × 10^−4^	0.6	1	26	6.5 × 10^4^
3 × 10^−6^	3 × 10^−4^	1 × 10^−5^	1 × 10^−5^	0.6	9.9	2.6	6.4 × 10^5^

This empirical calibration of the model is at best qualitative. However, the problem turns out to be rather constrained, such that it seems to inevitably lead to a high mutation rate and a strong dBGC. Thus, for instance, allowing for a lower *g*, under otherwise fixed values for *u* and *α*, would lead to equilibrium levels of erosion that are low compared to empirically observed levels (*H* > 80%, table 1, second row). Decreasing *g* could be compensated for by also decreasing *u* by the same factor, so as to predict levels of depletion of the order of 50% (third row of table 1). However, this would then lead to insufficient levels of PRDM9 diversity, essentially pushing the process into the succession regime (*D* = 1). Alternatively, compensating the decrease in *g* by a decrease in the value of *α* would push the Red Queen into the weak selection regime in the mouse (fourth row *S*_0_ = 2.6).

Finally, our estimates of *u* and *g* can be compared with current available empirical evidence. Concerning *u*, based on sperm-typing experiments, a mutation rate of the order of 10^−5^, thus not so far from our own estimate, has been inferred in humans [28]. However, this reported value corresponds to the raw mutation rate, i.e. not corrected for the production of non-functional, deleterious or redundant PRDM9 variants recognizing similar target sites. Here, by contrast, *u* is the *functional* mutation rate, leading to new functional alleles with essentially new targets across the genome, which is expected to be substantially lower than the raw mutation rate.

Concerning the strength of dBGC, based on a high-resolution genetic map of a fraction of chromosome 1 in the mouse [31], the mean rate of CO across hot spots can be estimated to be of the order of 10^−3^. Assuming a 10 : 1 ratio for NCO versus CO events, this gives an estimate for *g* of the order of 10^−2^, thus slightly higher than our estimate. However, this is an upper bound, granting systematic conversion of the target site of PRDM9 upon each DSB initiation and assuming complete inactivation of the target site by single point mutations. In practice, there is indirect evidence that conversion of the target site is not systematic in the mouse, in particular in the case of NCOs [32]. In addition, mutations are only partially inactivating PRDM9 binding, as indicated by the presence of multiple substitutions at many of the now extinct hot spots. Combined together, these two lines of empirical observations suggest that *g* might in fact be substantially lower than 10^−2^.

Thus, altogether, the strength of dBGC and the functional mutation rate at the PRDM9 locus are both predicted by our model to be close to their highest possible value compatible with current empirical observations.

## 6. Discussion

In this work, we have presented a combination of simulations and analytical and numerical approximations, giving new insights into the population genetics of the Red Queen model of recombination. Our more specific aim was to achieve a better understanding of how the interplay between mutational input, positive selection and dBGC modulates the statistical patterns of PRDM9 diversity and the dynamics of recombination landscapes. In the end, our analysis yields a global picture of the qualitative regimes and the scaling behaviour of the Red Queen model, as a function of effective population size and the parameters of the genetic system.

### (a) Evolutionary regimes and scaling behaviour

The stationary regime of the Red Queen process can be characterized in terms of the mean recombination activity *R* (or, alternatively, the mean fraction of non-eroded targets *H*), genetic diversity at the PRDM9 locus *D*, characteristic turnover time of recombination landscapes *T* and strength of selection acting on new PRDM9 alleles *s*_0_. For the most part, the overall scaling behaviour of all of these quantities depends on the parameters of the model only through two key compound parameters: first, the scaled mutation rate *μ* = 4*N*_e_*u* at the PRDM9 locus and, second, the parameter *ε* = *ρ*/*μ*, which measures the relative strength of the two arms of the Red Queen (erosion, at rate *ρ* = 4*N*_e_*vg*, versus restoration of recombination, at rate *μ* = 4*N*_e_*u*) and thus determines the erosion–restoration balance of the Red Queen. The scaling relations are captured in a particularly compact form in the weak erosion regime, using a first-order approximation as a function of *ε*. This approximation is less accurate in more intense regimes, although, even then, it correctly describes the qualitative behaviour of the process.

First, the mean level of erosion *R*, strength of selection on new PRDM9 alleles *s*_0_ and turnover time *T* do not depend on effective population size *N*_e_ (figures 2 and 4). Intuitively, the equilbrium recombination activity is determined by the relative magnitude of the two forces of the Red Queen, erosion and invasion, both of which are proportional to *N*_e_. The effective population size cancels out from their ratio, and thus the equilibrium set point does not depend on *N*_e_.

Second, both 1 − *R* and *s*_0_ increase with the erosion rate *vg* and decrease with the mutation rate *u* at the PRDM9 locus. Similar observations concerning the role of mutation rates at the targets and at the PRDM9 locus were previously reported based on simulation analyses [21]. Here, we give a more quantitative estimate of this scaling, in terms of *ε*. Specifically, in the weak erosion regime, 1 − *R* and *s*_0_ scale as 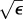, whereas *T* scales as 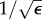.

Third, in the polymorphic regime, mean recombination activity and PRDM9 diversity are essentially uncoupled—in the weak erosion limit, the first depends only on *ε* and the second only on *N*_e_*u*. Intuitively, the mean recombination activity implied by a given value of *ε* can be equivalently realized, either by few PRDM9 alleles segregating at high frequencies, eroding their targets at a high rate and thus quickly replaced by other alleles, or by many alleles segregating at low frequencies, eroding their targets at a lower rate and replaced less often. The choice between these alternative regimes is essentially determined by the mutational input at the PRDM9 locus, *N*_e_*u*—thus, in a sense, neutrally.

This last point is of particular importance concerning the interpretation of the empirically observed patterns of polymorphism and divergence at the PRDM9 locus [17–20]. Positive selection on PRDM9 may easily reach very high levels under reasonable parameter regimes of the Red Queen process, which will easily translate into high ratios of non-synonymous to synonymous rates. Even when very strong, however, this type of positive selection is not diversifying. At least under the assumptions of the models explored here, mutation, not selection, explains the intra-specific genetic diversity of PRDM9.

The simulations presented here are based on relatively simple (exponential and power-law) fitness schemes. In practice, the fitness of individuals as a function of their PRDM9 genotype might certainly be more complicated. In particular, it may not necessarily be monotonic and instead show a maximum for an intermediate CO rate. Further work might be needed in this direction. Nevertheless, many of our results appear to be relatively general. In particular, the scaling as a function of *N*_e_ and the fact that diversity is determined by the mutational input are essentially valid under arbitrary fitness models, as long as the absolute fitness of a diploid can be expressed as a function of the activities (fraction of active targets) of its two alleles. On the other hand, more complex models could be imagined, for which the results presented here are not valid, in particular, if the fitness of an allele directly depends on population-level quantities, such as the amount of linkage disequilibrium (see below).

Self-consistent mean-field approximations have already been used in the context of other evolutionary problems (e.g. in [33]). They could, in principle, be applied to many other evolutionary Red Queen models, at least if the aim is to characterize the stationary regime. A key argument, repeatedly used throughout our self-consistent mean-field derivation, is the tiling principle (figure 6). This argument is in fact a specific instance of the ergodicity principle, which states that averages over the population and averages over allelic trajectories have identical expectations at stationarity. The specific form taken here by this principle is a consequence of the strong-selection assumption. However, ergodicity arguments could be used under more general conditions, in combination with diffusion approximations, so as to derive mean-field approximations that would also be valid in nearly neutral regimes. Such mathematical developments appear to be relatively complex, although not out of reach.

### (b) Empirical adequacy of the model

Our tentative empirical calibration of the model based on data available for the mouse suggests a high mutation rate (*u* ∼ 3 × 10^−6^) and a strong dBGC (*g* ∼ 3 × 10^−3^). Compared to currently available empirical evidence, these estimates are not unreasonable, although perhaps too high. There are several possible reasons for this potential overestimation. One possibility would be that the levels of depletion of the order of 50% that we have used to fit the model have been measured specifically for the major PRDM9 alleles [25,26], which are precisely those alleles for which erosion is expected to be as its highest levels. By contrast, the erosion level predicted by the model, through the summary statistic *H*, is supposed to be a weighted average over all segregating alleles, thus potentially less extreme than 50% in the case of the mouse. Similarly, the raw diversity at the PRDM9 locus is potentially contributed at least in part by functionally equivalent alleles, recognizing similar target sites. By contrast, and like for the mutation rate *u*, the diversity *D* predicted by our model is supposed to correspond to the functional diversity (i.e. the effective number of functionally distinct alleles), thus potentially lower than the raw diversity. Accounting for these two factors could easily result in lower estimates for both *g* and *u*.

Finally, another possible explanation would be that, in our model, the fitness of an allele only depends on the total recombination activity induced by this allele, irrespective of the fine-scale distribution of recombination over the genome. This may be reasonable if the main role of recombination is to promote correct chromosome segregation. However, the fine-scale distribution of recombination across the genome plays an important role in shaping the distribution of LD. In particular, under low PRDM9 diversity, recombination is concentrated on few hot spots that are constantly reused at the level of the population, thus creating large haplotype blocks within which LD tends to accumulate, potentially resulting in a genetic load through background selection or interference between concurrent selective sweeps. In this context, rare PRDM9 alleles could be favoured because they break up those large haplotype blocks, thereby contributing to a more efficient dissipation of LD. Unlike what we have considered in our model thus far, this specific form of selection is inherently frequency-dependent and therfore genuinely diversifying. As a result, it would provide a mechanism for explaining high levels of PRDM9 polymorphism without having to invoke very high mutation rates at this locus. In turn, allowing for a lower mutation rate *u* would then make it possible to accomodate more reasonable values for the strength of dBGC, *g*, while still predicting significant levels of depletion of recombination landscapes.

### (c) Perspectives

In spite of the remaining uncertainty about the exact value of the mutation rate of the Zn-finger domain of PRDM9, there is no doubt that this rate is one of the highest among all protein-coding genes encoded by mammalian genomes. Given the important role played by the mutational input at the PRDM9 locus in the restoration of recombination in the face of ongoing erosion by dBGC, this empirical fact raises the question of why the key trans-acting factor of the Red Queen of recombination turnover happens to have such a high mutation rate in the first place. An interesting possibility would be that PRDM9 has been co-opted in this intra-genomic Red Queen precisely for that reason. Altermatively, the detailed genetic structure of the Zn-finger domain could have been progressively optimized through a higher-level evolutionary process. These interesting speculations could be further investigated in the context of the present modelling framework.

Finally, another intriguing aspect of PRDM9 is its role in hybrid sterility [34]. Recent developments have suggested that the hybrid sterility phenotype is directly related to the asymmetry in the patterns of PRDM9 binding along chromosomes of meiotic cells in F1 hybrids [25,26]. In turn, this asymmetry is a direct consequence of the differential erosion of PRDM9 targets along the two parental chromosomes, itself due to distinct major PRDM9 alleles segregating in the two subpopulations of origin [26]. Altogether, these observations point towards a potentially important role for the Red Queen process of recombination turnover in the creation of genetic barriers between subspecies. The modelling framework introduced here could certainly be extended in a meta-population context, so as to further investigate those particularly interesting questions.

## Supplementary Material

Appendices and Supplementary figures

## Data accessibility

The data used for this study have been deposited in https://github.com/ThibaultLatrille/RedQueen.
