# Supplementary material for: The Red Queen model of recombination hot-spot evolution: a theoretical investigation
Source: Philos Trans R Soc Lond B Biol Sci. 2017 Nov 6;372(1736):20160463. doi: 10.1098/rstb.2016.0463 (PMC5698625; doi:10.1098/rstb.2016.0463)
Supplement: Appendices and Supplementary figures [file rstb20160463supp1.pdf]

The Red-Queen model of recombination hot-spot evolution: a theoretical investigation.

Supplementary information

Thibault Latrille, Laurent Duret, Nicolas Lartillot

Laboratoire de Biométrie et Biologie Evolutive, Lyon, France.

nicolas.lartillot@univ-lyon1.fr **Running head:** The recombination Red-Queen

Appendix 1. Linearized mean-field derivation

We first derive an explicit soluton for x as a function of θ. Starting from the differential equations for x and θ

$$\begin{aligned}\frac{dx}{dt} &= \frac{f'(R)}{2f(R)}\left(\theta - R\right)x \\ \frac{d\theta}{dt} &= -\rho x\theta\end{aligned}$$

and combining them gives:

$$\frac{dx}{d\theta} = \frac{dx}{dt}/\frac{d\theta}{dt} = \frac{1}{\rho}\frac{f'(R)}{2f(R)}\left(\frac{R}{\theta} - 1\right)$$

which has solution

$$\begin{aligned}x &= \frac{1}{\rho}\frac{f'(R)}{2f(R)}\left[R\ln\theta - \theta\right]_0^{R_\infty} \\ &= \frac{f'(R)}{2\rho f(R)}(1 - \theta + R\ln\theta) + x_{init}\end{aligned}$$

or, in explicit notation as a function of t:

$$x_t = \frac{f'(R)}{2\rho f(R)}(1 - \theta_t + R\ln\theta_t) + x_{init}$$

When $t \to \infty$, θ_t converges to R_∞ and $x(\theta_t)$ to 0. In addition, since $x_{\text{init}} = 1/N_e$ is small, we can let it go to 0, which yields a relation between R and R_∞:

$$0 \simeq 1 - R_\infty + R\ln(R_\infty) \tag{1}$$

Second, using the tiling argument (figure 6 of the main text):

$$
\begin{aligned}
\tau &= \int_0^{+\infty} x dt \\
&= \int_1^{R_\infty} x \frac{dt}{d\theta} d\theta \\
&= \int_1^{R_\infty} x \left(\frac{d\theta}{dt}\right)^{-1} d\theta \\
&= -\int_1^{R_\infty} x \frac{1}{\rho x \theta} d\theta \\
&= -\int_1^{R_\infty} \frac{d\theta}{\theta} \\
&= -\frac{1}{\rho} \ln R_\infty \\
&= \frac{1 - R_\infty}{\rho R}
\end{aligned}
$$

where we have used equation 1 for the last step of the derivation.

A similar argument can be used for D, which can be obtained from:

$$
\sum_i x_i^2 \simeq \frac{1}{\tau} \int_0^{\infty} x_t^2 \mathrm{d}t,
$$

which is also analytically available, yielding:

$$
D = \left(\sum_i x_i^2\right)^{-1} = \frac{4\rho f(R)}{f'(R)\left[1 + R_\infty - 2R\right]}.
$$

Appendix 2. Generalized mean-field derivation

From equation 1 of the main text, the activity of the allele decays at a rate proportional to the allele's frequency:

$$\frac{d\theta}{dt} = -\rho x \theta.$$

This equation has general solution:

$$\theta_t = e^{-\rho \int_0^t x_u du} = e^{-\rho \xi_t} \tag{2}$$

where we define

$$\xi_t = \int_0^t x_u du.$$

The variable ξ can be seen as the *intrinsic age* of the allele. It accounts for the fact that an allele ages more rapidly if it segregates at a higher frequency. What equation 2 tells us is that the activity of the allele at a given time point only depends on the allele's intrinsic age. We can therefore parameterize all quantitites associated with a PRDM9 allele directly as a function of ξ, thus dispensing us with the need to solve x_t as a function of time.

Instead of the intrinsic age ξ, it is in fact more convenient to reparameterize the problem in terms of the dimensionless variable $z = \rho\xi$ (relative age). Since

$$\tau = \int_0^\infty x(u)du = \xi(\infty),$$

$z(t)$ is a monotonous function of time, varying from 0 to $\rho\tau$. The inverse of this function is noted $t^*(z)$. In the following, all quantities expressed in the z-parameterization will be indicated by a star. In particular, we define:

$$x^*(t) = x(t^*(z))$$

and

$$\theta^*(z) = \theta(z(t)) = e^{-z} \tag{3}$$

In the z-parameterization, the activity of a new-born allele is thus $\theta^*(0) = 1$, and the activity of a dying allele is equal to $\theta_\infty = \theta^*(\rho\tau) = e^{-\rho\tau}$. Other quantities will be reparameterized below as a function of z.

Some elementary relations between the t- and z-parameterizations can be pointed out, which will be useful in the following. First,

$$\frac{dz}{dt} = \rho x$$

and second

$$\frac{dx^*}{dz} = \frac{dx}{dt} \Big/ \frac{dz}{dt} = sx/\rho x = \frac{s^*(z)}{\rho}$$

where we have defined $s^*(z) = s(\theta(t))$, the selection coefficient associated with the allele, in the z-parameterization.

Second, the tiling principle (figure 6 of the main text) suggests that the mean time between successive invasions is approximately equal to:

$$\tau = \int_0^\infty x_u = \xi_\infty,$$

which reveals that τ can also be interpreted as the mean intrinsic longevity of PRDM9 alleles. This identity is merely a constraint implied by the stationarity of the process under constant population size. If, over its lifespan, each PRDM9 allele contributes a total population of $2N_e\xi_\infty$, then, in order for the total population size to remain constant, new alleles should invade the population once every ξ_∞ generations on average. Thus, $\xi_\infty = \tau$ (or equivalently, $z_\infty = \rho\tau$). In the succession regime, this constraint is trivially fulfilled by each successive allele being at a frequency of 1 during a total time τ. In the polymorphic regime, on the other hand, each allele's contribution is diluted out over a longer time period, overlapping the lifespan of other younger and older alleles. An analogy can be made here with an assembly line, of which we can vary the latency under the constraint of a constant total output.

Another interpretation of the tiling argument is as follows. Suppose we randomly sample an allele at stationarity. Then, the probability that this allele has intrinsic age ξ is uniformly distributed between 0 and τ. Equivalently, z is uniformly distributed between 0 and $\rho\tau$. As a consequence, we can approximate any average over the population, at stationarity, as an integral over z. For instance, the mean recombination activity is equal to:

$$R = \sum_i x_i \theta_i \quad \simeq \quad \frac{1}{\rho\tau}\int_0^{\rho\tau} \theta^*(z)dz = \frac{1}{\rho\tau}\int_0^{\rho\tau} e^{-z}dz = \frac{1-e^{-\rho\tau}}{\rho\tau}. \tag{4}$$

Using this trick, we can find simple integral expressions for all summary statistics that are averages over the population at stationarity. That is, for any function $q(x,\theta)$:

$$\begin{aligned} \sum_i x_i q(x_i, \theta_i) \quad &\simeq \quad \frac{1}{\tau}\int x(t)q(x(t), \theta(t))dt \\ &= \quad \frac{1}{\rho\tau}\int_0^{\rho\tau} q(x^*(z), \theta^*(z))dz \end{aligned}$$

Using this identity, we can express several key summary statistics, all of which are averages over the population at stationarity. Thus, the mean recombination activity is given by:

$$R = \sum_i x_i \theta_i \quad = \quad \frac{1}{\rho\tau}\int_0^{\rho\tau} \theta^*(z)dz = \frac{1}{\rho\tau}\int_0^{\rho\tau} e^{-z}dz = \frac{1-e^{-\rho\tau}}{\rho\tau}$$

The diversity:

$$D^{-1} = \sum_i x_i^2 = \frac{1}{\rho\tau}\int_0^{\rho\tau} x^*(z)dz = -\frac{1}{\rho^2\tau}\int_0^{\rho\tau} s^*(z)zdz$$

where we have integrated by parts, using the relation $dx^*/dz = s^*(z)/\rho$. Since the turnover time is equal to $T = \tau D$, this can also be rewritten as:

$$T^{-1} = (\tau D)^{-1} \quad = \quad -\frac{1}{(\rho\tau)^2}\int_0^{\rho\tau} s^*(z)zdz$$

The fitness of an allele of activity $\theta_0 = \theta^*(z_0)$ can be calculated by averaging over all possible diploid backgrounds:

$$w^*(z_0) = w(\theta_0) = \sum_i x_i f\left(\frac{\theta_0 + \theta_i}{2}\right) \quad = \quad \frac{1}{\rho\tau}\int_0^{\rho\tau} f\left(\frac{\theta^*(z_0) + \theta^*(z)}{2}\right)dz$$

and the mean fitness over the population is obtained by a double averaging procedure:

$$\bar{w} = \frac{1}{\rho\tau}\int_0^{\rho\tau} w^*(z)dz = \frac{1}{(\rho\tau)^2}\int_0^{\rho\tau}\int_0^{\rho\tau} f\left(\frac{\theta^*(z_1)+\theta^*(z_2)}{2}\right)dz_1dz_2$$

From these equations, the selection coefficient acting on an allele of age z, noted $s^*(z)$, is given by:

$$s^*(z) = \frac{w^*(z)-\bar{w}}{\bar{w}}$$

and in particular, the selection coefficient for a newborn allele is:

$$s_0 = s^*(0) = \frac{w^*(0)-\bar{w}}{\bar{w}}$$

The quantities R, T, w^* and s^* only depend on the fitness function f and the compound parameter $\rho\tau$. Thus, we can express s_0 more compactly as:

$$s_0 = H(\rho\tau).$$

In turn, τ, is the inverse of the rate of invasion of the population by new alleles:

$$\tau^{-1} = \lambda = 4N_e u s_0,$$

or, equivalently,

$$\rho\tau = \frac{4N_e vg}{4N_e u s_0} = \frac{vg}{us_0} \quad (5)$$

Using this relation, the self-consistent mean-field argument can be expressed directly on the selection coefficient s_0:

$$s_0 = H(\frac{\epsilon}{s_0})$$

The solution is found by a combination of Simpson's approach to numerically evaluate the integral function H, and Brent's method to find the self-consistent solution. This solution for s_0 can then be plugged into equation 5, yielding the value of $\rho\tau$. Finallly, all summary statistics of interest, as integrals depending on $\rho\tau$, can now be computed either analytically or numerically, depending on the statistic.

Appendix 3. Varying recombination rates across hot spots

This version of the model assumes that the distribution of relative recombination rates across the hot spots associated with a new born PRDM9 allele is a gamma distribution, of mean 1 and shape parameter a (with smaller value of a inducing higher variance across hot spots):

$$\phi(c) = \frac{a^a}{\Gamma(a)} c^{a-1} e^{-ac}.$$

Consider a typical PRDM9 allele. For this allele, we define $\theta_c(t)$ as the fraction of hot spots with recombination rate c that are active at time t. The rate of erosion for this specific subset of hot spots is given by:

$$\frac{d\theta_c}{dt} = -\rho c x \theta.$$

By a similar argument as the one used in the last section, this has solution:

$$\theta_c(t) = e^{-\rho c \xi(t)} = e^{-cz(t)}$$

or, more directly in the z-parameterization:

$$\theta_c^*(z) = e^{-cz}$$

The total (relative) recombination activity induced by a PRDM9 allele with intrinsic age z is then obtained by averaging θ_c^* over c (under the gamma), which is analytical:

$$\theta^*(z) = \int c\theta_c^*(z)\phi(c)dc = \left(\frac{a}{a+z}\right)^{a+1} \tag{6}$$

From there, the overall derivation is essentially the same as in the previous section, with the only difference that the integral equations for w and s_0 are now computed using the expression for $\theta^*(z)$ given by equation 6, instead of equation 3. These expressions are, as before, functions of $\rho\tau = \frac{ug}{vs_0}$, again giving a self-consistent relation on s_0 which can be solved numerically.

As before, once the self-consistent solution is found, the summary statistics at the equilibrium set-point can be computed by averaging over z. In particular, the mean recombination activity over the population is now:

$$R = \frac{1}{\rho\tau}\int_0^{\rho\tau} \theta^*(z)dz \quad = \quad \frac{1}{\rho\tau}\left[1 - \left(\frac{a}{a+\rho\tau}\right)^a\right] \tag{7}$$

In addition to the mean recombination rate R, the mean fraction of active targets per allele, which we call H, is another statistic of interest, for which direct empirical evidence is available (Baker *et al.*, 2015; Davies *et al.*, 2016). Thus far, without variance across hot spots in their basal recombination rate, the two statistics R and H were identical. However, this is not anymore the case under the gamma model now considered. The fraction of active targets for an allele of age z, $h^*(z)$ is the mean of $\theta_c(z)$ over the gamma distribution:

$$h^*(z) \quad = \quad \int \theta_c^*(z)\phi(c)dc = \left(\frac{a}{a+z}\right)^a$$

and the average over the population is, again, obtained by averaging over z :

$$H = \frac{1}{\rho\tau}\int_0^{\rho\tau} h^*(z)dz \tag{8}$$

This is equal to:

$$H \quad = \quad \frac{1}{\rho\tau}\frac{a}{(a-1)}\left[1 - \left(\frac{a}{a+\rho\tau}\right)^{a-1}\right]$$

for $a \neq 1$, and to:

$$H \quad = \quad \frac{1}{\rho\tau}\ln(1+\rho\tau)$$

for $a = 1$.

Appendix 4. First-order development under the weak-erosion regime

To derive the small recombination gap approximation, we start by recalling the integral expression for the fitness of an allele of activity $\theta^*(z_0)$:

$$w^*(z_0) \quad = \quad \frac{1}{\rho\tau}\int_0^{\rho\tau} f\left(\frac{\theta^*(z_0)+\theta^*(z)}{2}\right) dz$$

Then, defining

$$\alpha \quad = \quad |f'(0)(\theta^*)'(0)|$$

making a first-order Taylor development of f at 0:

$$f\left(\frac{\theta^*(z_0)+\theta^*(z)}{2}\right) \quad \simeq \quad f(0) - \frac{\alpha}{2}(z_0+z) = 1 - \frac{\alpha}{2}(z_0+z)$$

and integrating with respect to z gives an approximation for $w^*(z_0)$:

$$w^*(z_0) \quad \simeq \quad \frac{1}{\rho\tau}\int_0^{\rho\tau} 1 - \frac{\alpha}{2}(z_0+z)dz = 1 - \frac{\alpha z_0}{2} - \frac{\alpha\rho\tau}{4}$$

Similarly, the mean fitness over the population is approximately equal to:

$$\begin{aligned}
\bar{w} \quad &= \quad \frac{1}{\rho\tau}\int_0^{\rho\tau} w^*(z)dz = \frac{1}{(\rho\tau)^2}\int_0^{\rho\tau}\int_0^{\rho\tau} f\left(\frac{\theta^*(z_1)+\theta^*(z_2)}{2}\right) dz_1 dz_2 \\
&\simeq \quad \frac{1}{(\rho\tau)^2}\int_0^{\rho\tau}\int_0^{\rho\tau} f\left(1 - \frac{\alpha}{2}(z_1+z_2)\right) dz_1 dz_2 \\
&= \quad 1 - \frac{\alpha\rho\tau}{2}
\end{aligned}$$

The selection coefficient can then be calculated:

$$s^*(z_0) = \frac{w^*(z_0) - \bar{w}}{\bar{w}} \quad \simeq \quad \frac{\alpha\rho\tau}{4} - \frac{\alpha z_0}{2}$$

In particular, for $z_0 = 0$:

$$s^*(0) = \frac{w^*(0) - \bar{w}}{\bar{w}} \quad = \quad \frac{\alpha\rho\tau}{4} = \frac{\alpha v g}{4us^*(0)}$$

or, equivalently

$$s^*(0)^2 \quad = \quad \frac{\alpha\rho\tau}{4} = \frac{\alpha v g}{4u}$$

which yields an explicit solution for $s^*(0)$:

$$s^*(0) \;=\; \frac{1}{2}\sqrt{\frac{\alpha v g}{u}} = \frac{1}{2}\sqrt{\alpha\epsilon} \propto \sqrt{\epsilon}$$

This can then be used to get an explicit expression for $\rho\tau$:

$$\rho\tau = \frac{vg}{us^*(0)} \;=\; \frac{vg}{u}\sqrt{\frac{4u}{\alpha v g}} = 2\sqrt{\frac{vg}{u\alpha}} = 2\sqrt{\frac{\epsilon}{\alpha}} \propto \sqrt{\epsilon}$$

and for R:

$$R = \frac{1 - e^{-\rho\tau}}{\rho\tau} \;\simeq\; 1 - \frac{1}{2}\rho\tau = 1 - \sqrt{\frac{vg}{u\alpha}} = 1 - \sqrt{\frac{\epsilon}{\alpha}}$$

and thus

$$1 - R \;\propto\; \sqrt{\epsilon}$$

Concerning D, the integral expression is:

$$D^{-1} \;=\; -\frac{1}{\tau}\frac{1}{\rho\tau}\int_0^{\rho\tau} s^*(z) z dz$$

which, using the approximation given above for $s^*(0)$ and after some algebra gives:

$$D^{-1} = \frac{s^*(0)}{6\tau}$$

Noting that $\tau = 4Nus^*(0)$, this yields a surprisingly simple scaling identity for D:

$$D \;\simeq\; 24N_e u$$

Finally,

$$T = D\tau = D\rho\tau/\rho \simeq \frac{24N_e u}{4N_e v g} 2\sqrt{\frac{vg}{u\alpha}} = 12\sqrt{\frac{u}{vg\alpha}} = 12\frac{1}{\sqrt{\alpha\epsilon}} \propto \frac{1}{\sqrt{\epsilon}}$$

References

Baker, C. L., Kajita, S., Walker, M., Saxl, R. L., Raghupathy, N., Choi, K., Petkov, P. M. & Paigen, K. 2015 PRDM9 drives evolutionary erosion of hotspots in Mus musculus through haplotype-specific initiation of meiotic recombination. *PLoS Genet.*, **11**(1), e1004 916.

Davies, B., Hatton, E., Altemose, N., Hussin, J. G., Pratto, F., Zhang, G., Hinch, A. G., Moralli, D., Biggs, D. *et al.* 2016 Re-engineering the zinc fingers of PRDM9 reverses hybrid sterility in mice. *Nature*, **530**(7589), 171–176.

Supplementary Figures

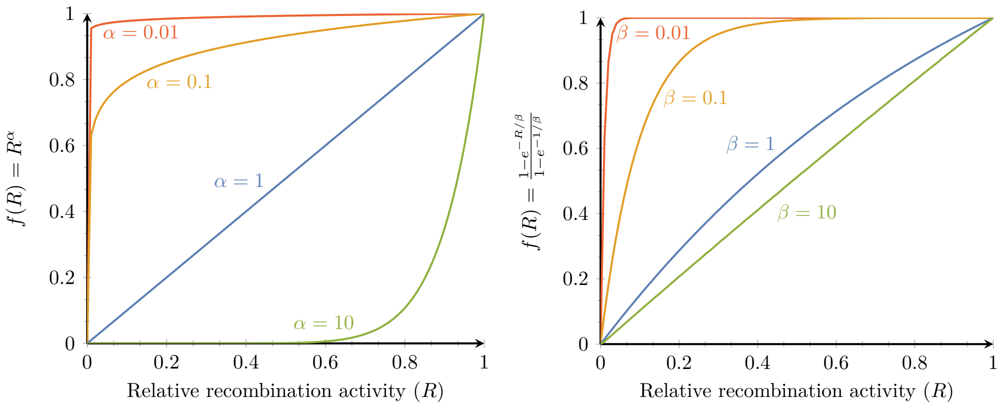

Figure S1. Fitness functions $f(R)$ used in this work. Power-law (left) and exponential (right) models.

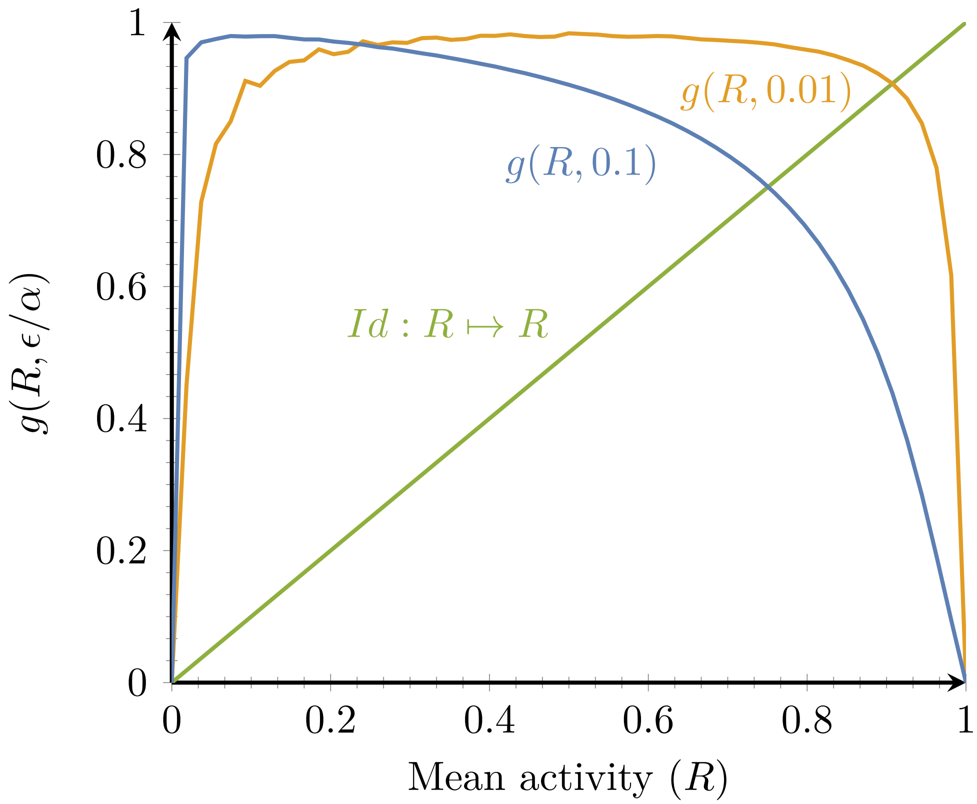

Figure S2. Self-consistent linearized mean-field solution as a fixed point, in the form $R = g(R, \epsilon/\alpha)$, for two values of $\epsilon/\alpha = \frac{vg}{\alpha u}$, under the power-law fitness function.

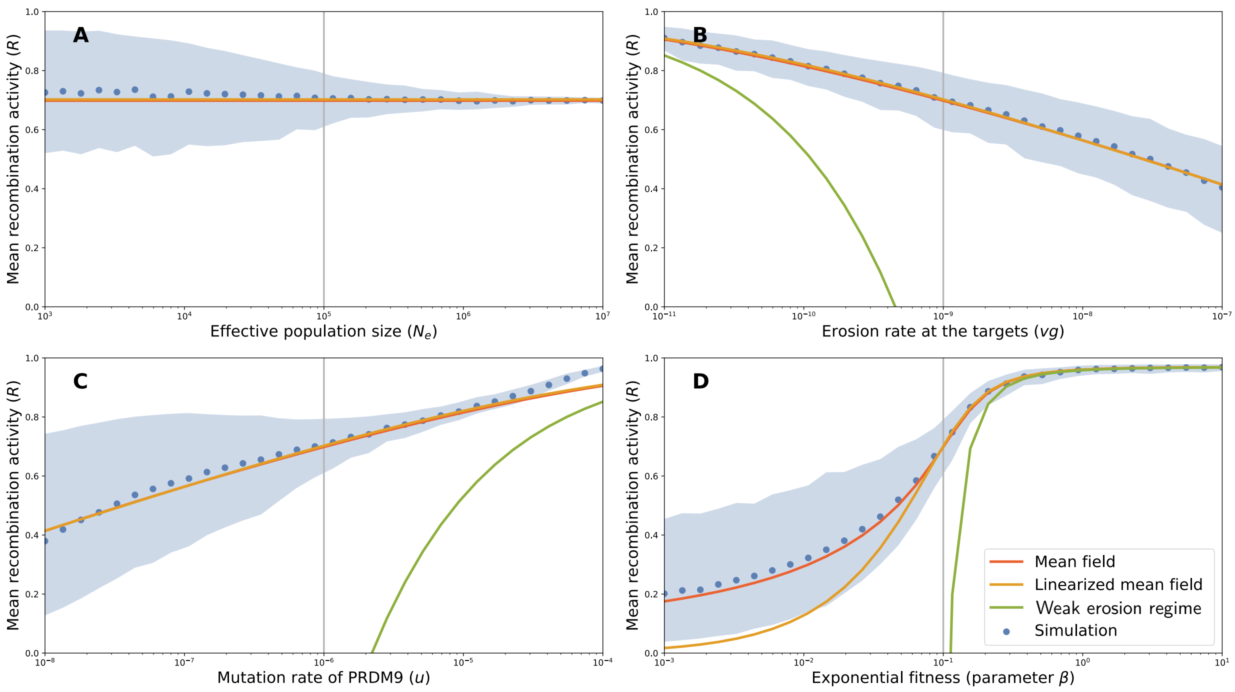

Figure S3. Mean recombination activity R, as a function of effective population size N_e (A), erosion rate vg (B), mutation rate at the PRDM9 locus u (C) and the fitness parameter β (D) under the exponential fitness model. Mean-field approximations, either linearized (orange) or generalized (red) and weak erosion approximation (green) are shown on the top of the mean and variance over the simulations (blue). For plots under the power-law fitness model, see Figure 2.

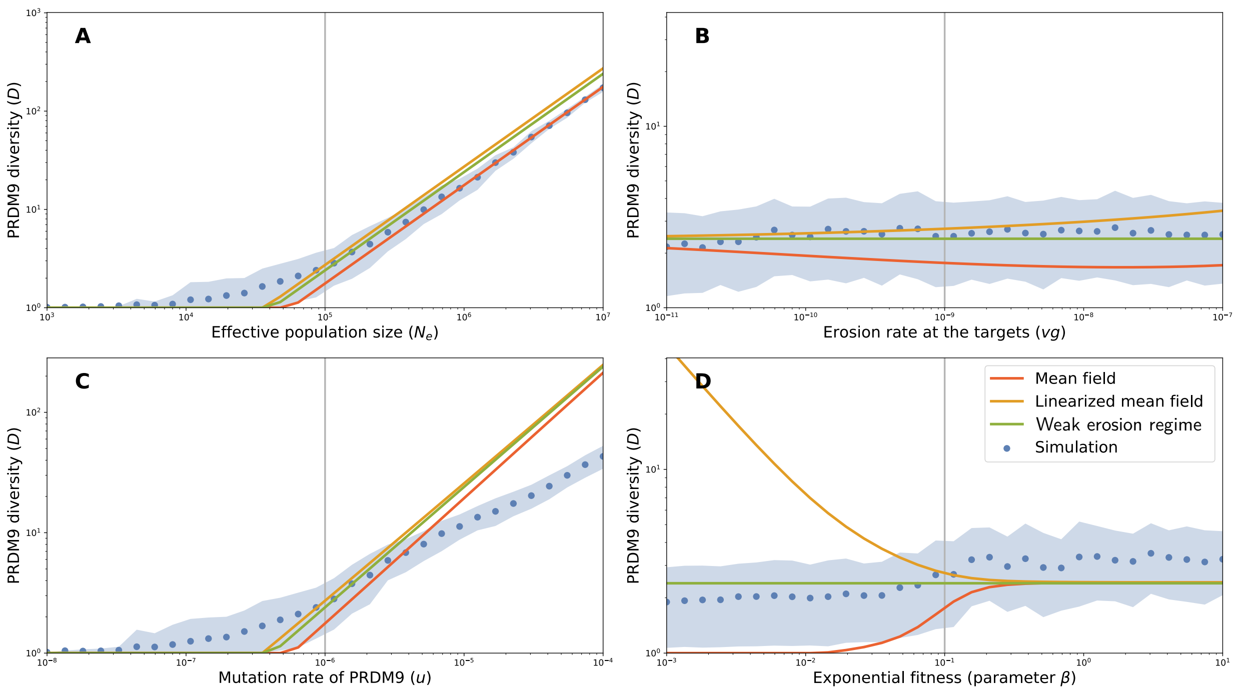

Figure S4. Genetic diversity at the PRDM9 locus D, as a function of effective population size N_e (A), erosion rate vg (B), mutation rate at the PRDM9 locus u (C) and the fitness parameter β (D) under the exponential fitness model. Mean-field approximations, either linearized (orange) or generalized (red) and weak erosion approximation (green) are shown on the top of the mean and variance over the simulations (blue). For plots under the power-law fitness model, see Figure 3.

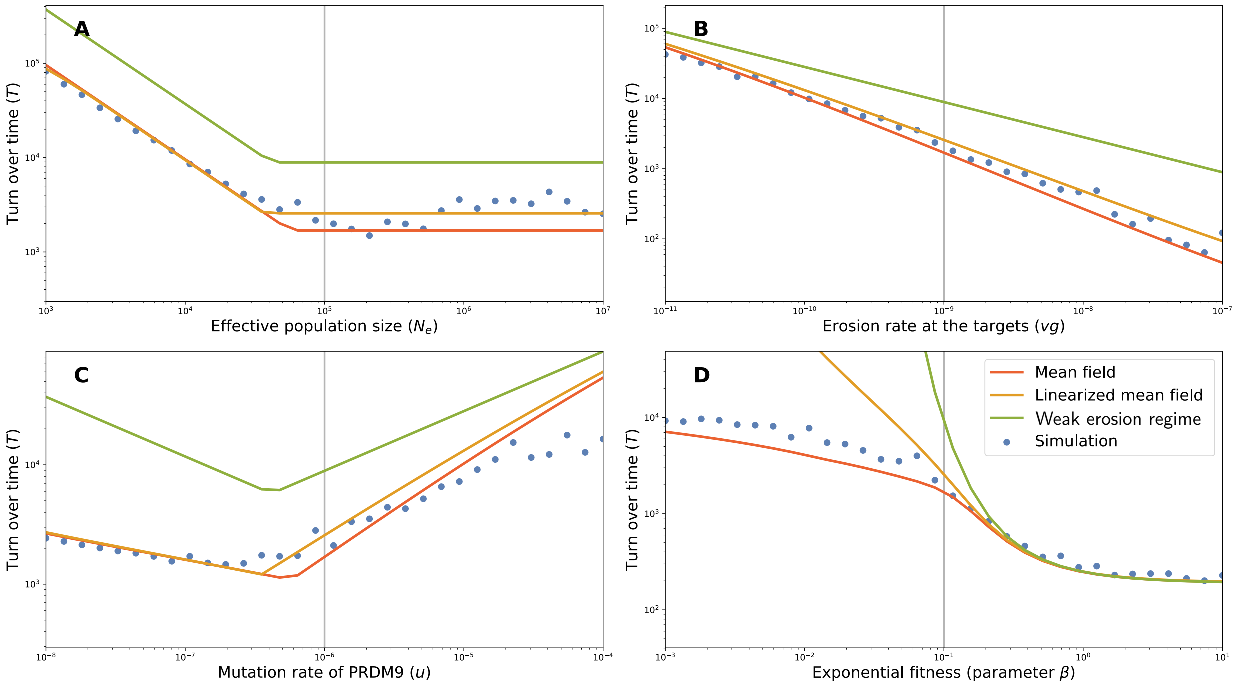

Figure S5. Turnover time T, as a function of effective population size N_e (A), erosion rate vg (B), mutation rate at the PRDM9 locus u (C) and the fitness parameter β (D) under the exponential fitness model. Mean-field approximations, either linearized (orange) or generalized (red) and weak erosion approximation (green) are shown on the top of the mean and variance over the simulations (blue). For plots under the power-law fitness model, see Figure 4.

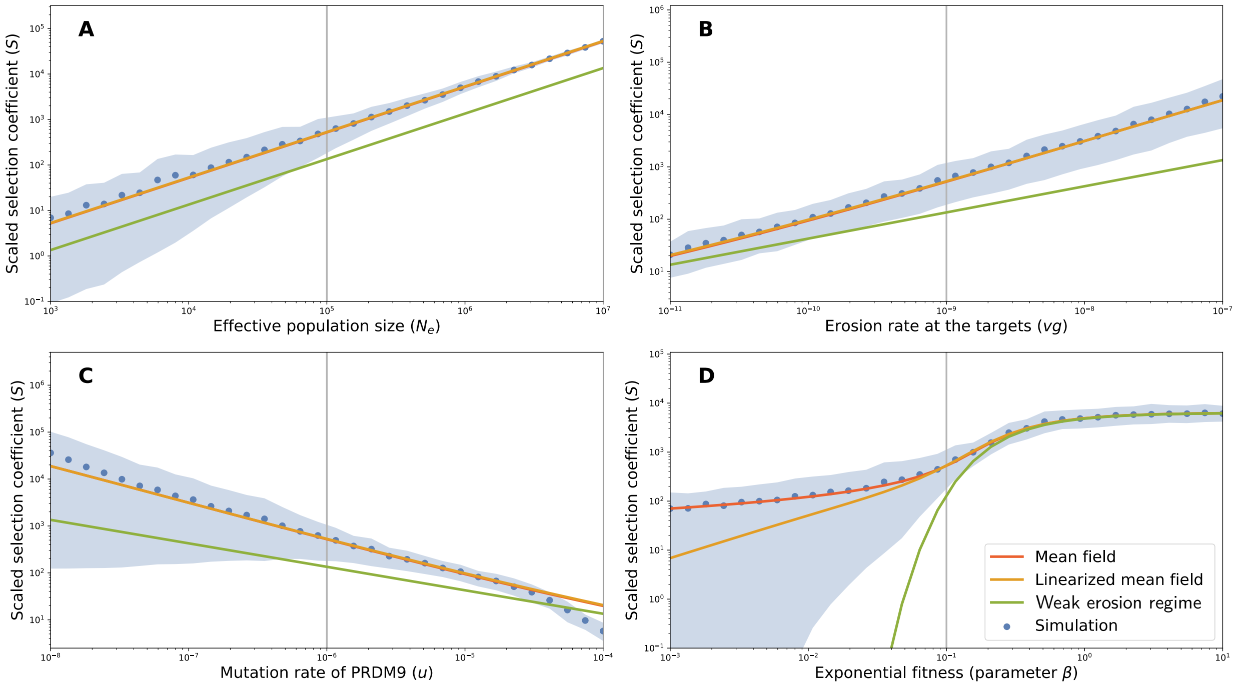

Figure S6. Scaled selection coefficient $S = 4N_e s_0$ associated with a new PRDM9 allele entering the population, as a function of effective population size N_e (A), erosion rate vg (B), mutation rate at the PRDM9 locus u (C) and the fitness parameter β (D) under the exponential fitness model. Mean-field approximations, either linearized (orange) or generalized (red) and weak erosion approximation (green) are shown on the top of the mean and variance over the simulations (blue).

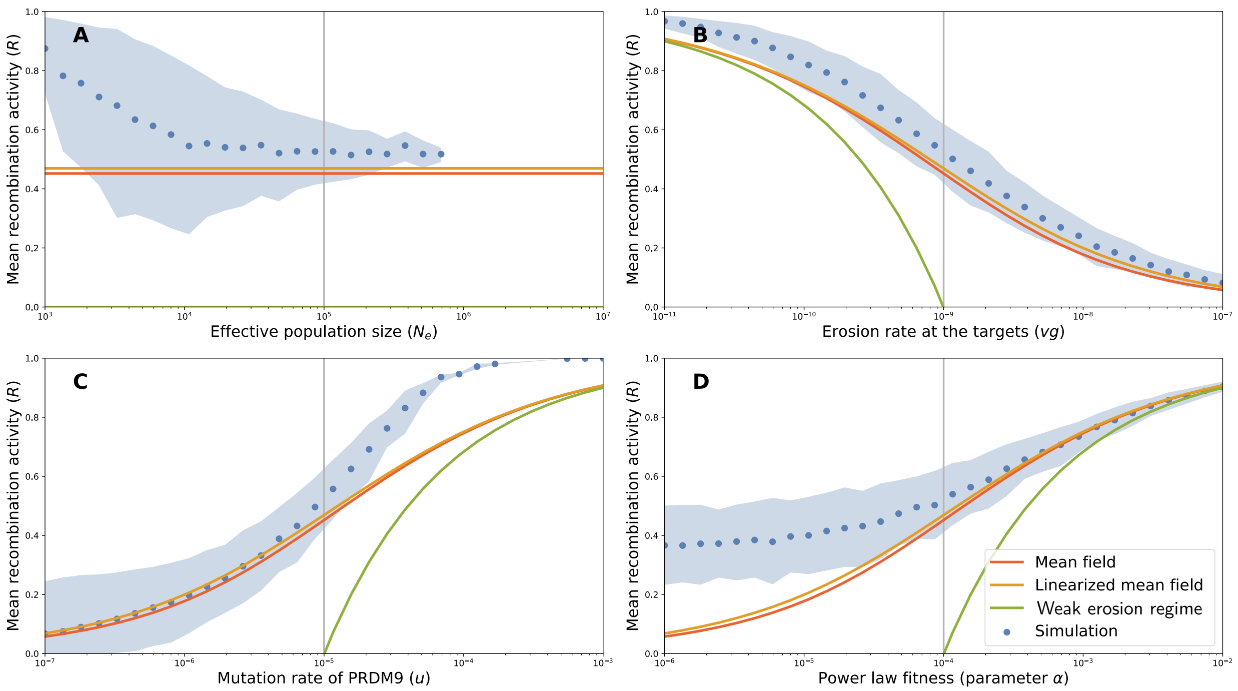

Figure S7. Mean recombination activity R, as a function of effective population size N_e (A), erosion rate vg (B), mutation rate at the PRDM9 locus u (C) and the fitness parameter α (D) under the power-law fitness model, under weak selection. Mean-field approximations, either linearized (orange) or generalized (red) are shown on the top of the mean and variance over the simulations (blue).

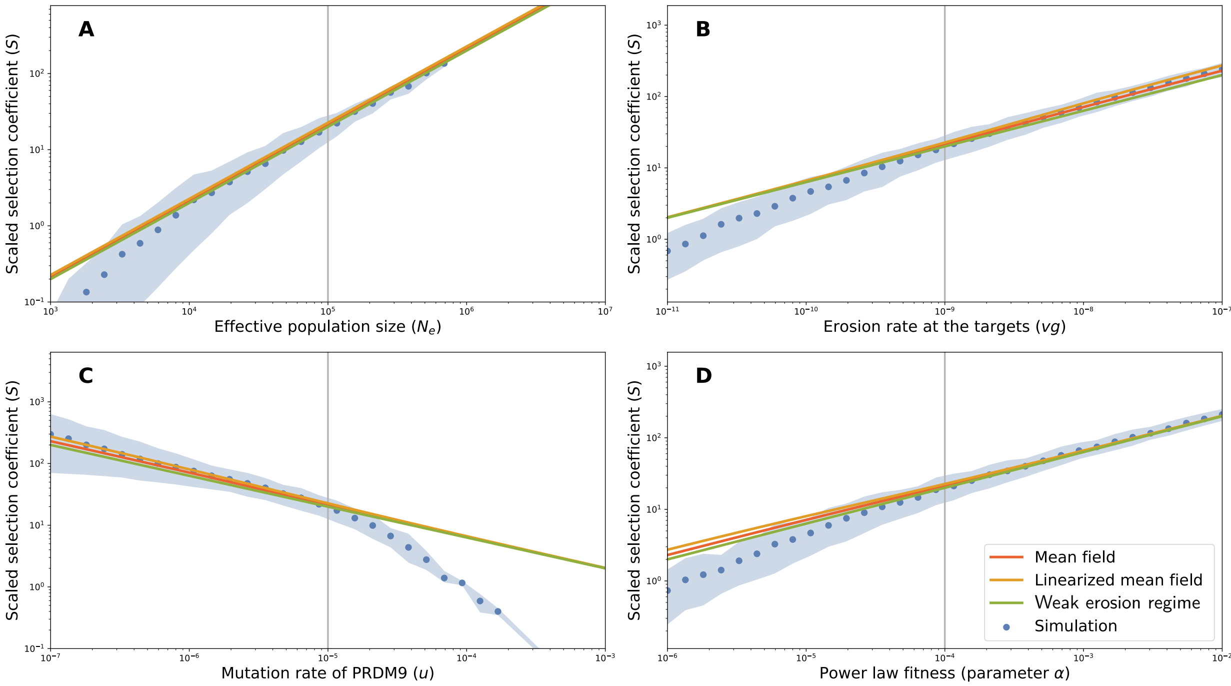

Figure S8. Scaled selection coefficient $S = 4N_e s_0$ associated with a new PRDM9 allele entering the population, as a function of effective population size N_e (A), erosion rate vg (B), mutation rate at the PRDM9 locus u (C) and the fitness parameter α (D) under the power-law fitness model, under weak selection. Mean-field approximations, either linearized (orange) or generalized (red) are shown on the top of the mean and variance over the simulations (blue).

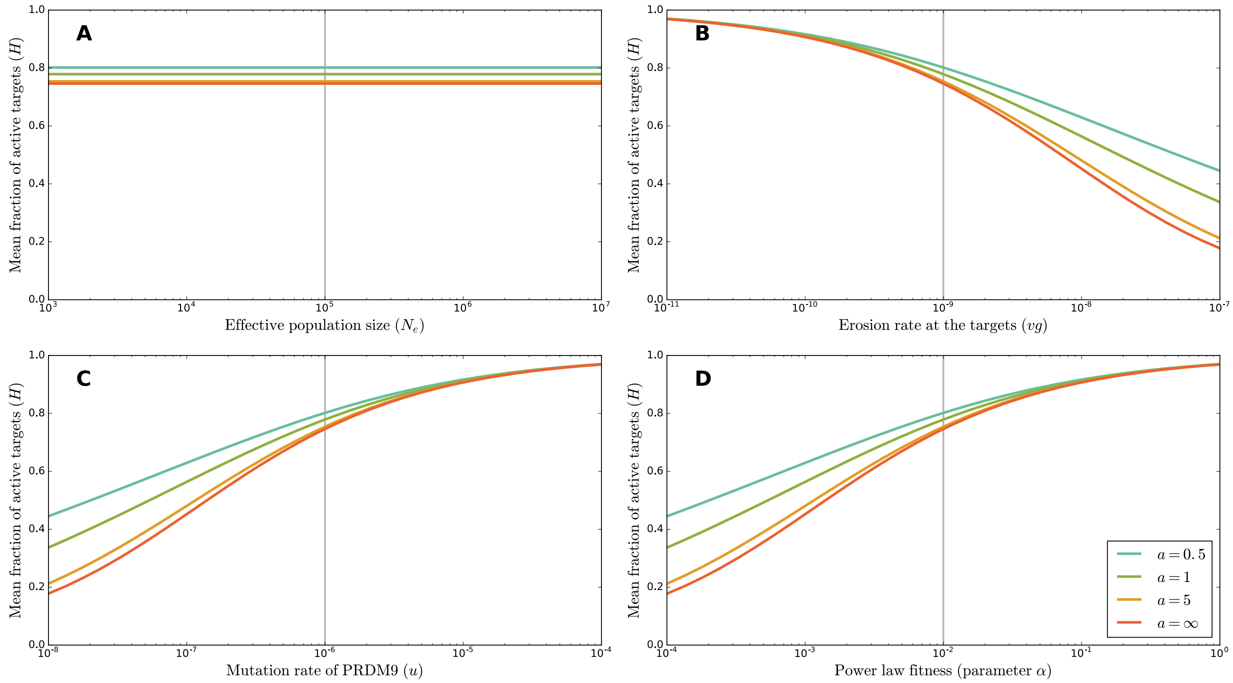

Figure S9. Mean fraction of active targets H, calculated under the generalized mean-field approximation, as a function of effective population size N_e (A), erosion rate vg (B), mutation rate at the PRDM9 locus u (C) and the fitness parameter α (D) under the power-law fitness model and with variance in hot spot strength, with shape parameter $a = 0.5$, 1, 5 and $+\infty$.

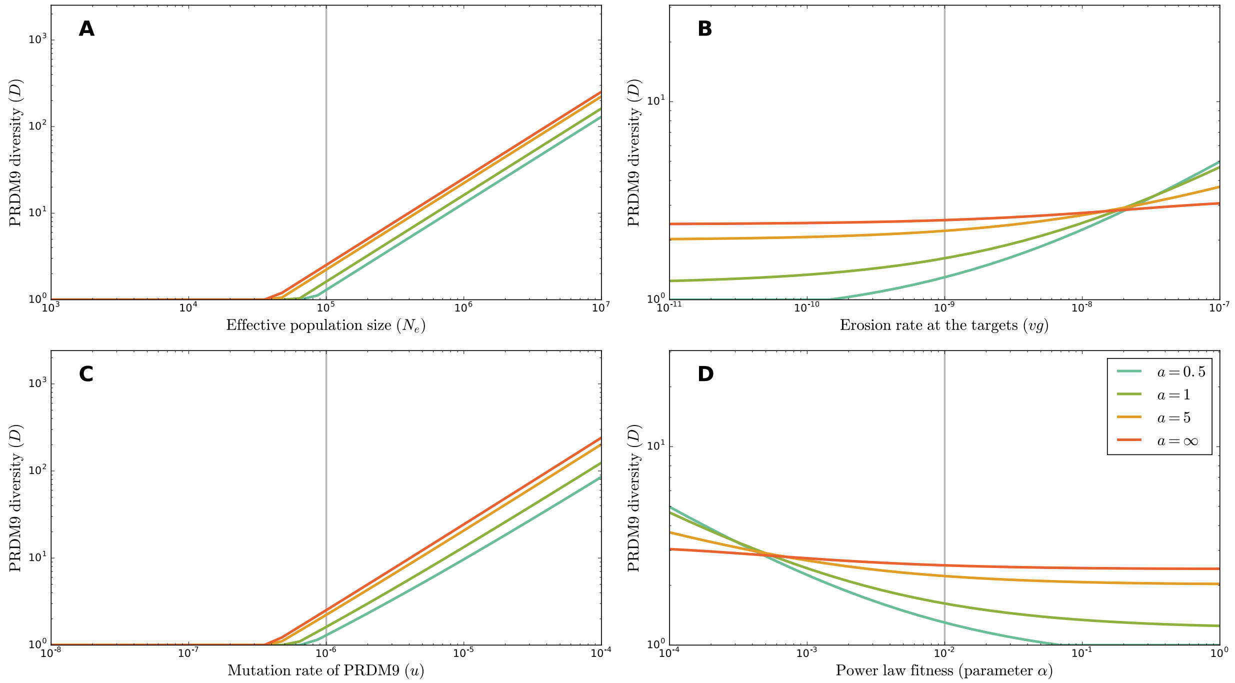

Figure S10. Genetic diversity at the PRDM9 locus D, calculated under the generalized mean-field approximation, as a function of effective population size N_e (A), erosion rate vg (B), mutation rate at the PRDM9 locus u (C) and the fitness parameter α (D) under the power-law fitness model and with variance in hot spot strength, with shape parameter $a = 0.5$, 1, 5 and $+\infty$.

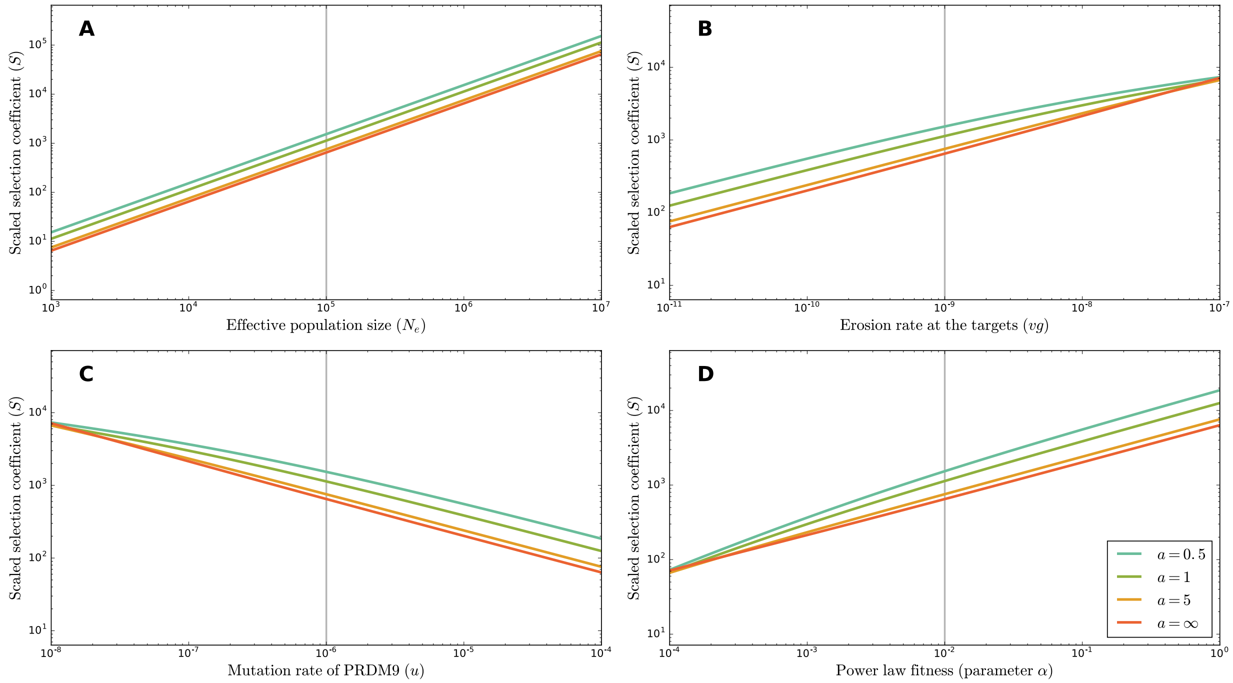

Figure S11. Scaled selection coefficient $S = 4N_e s_0$ associated with a new PRDM9 allele entering the population, calculated under the generalized mean-field approximation, as a function of effective population size N_e (A), erosion rate vg (B), mutation rate at the PRDM9 locus u (C) and the fitness parameter α (D) under the power-law fitness model and with variance in hot spot strength, with shape parameter $a = 0.5$, 1, 5 and $+\infty$.

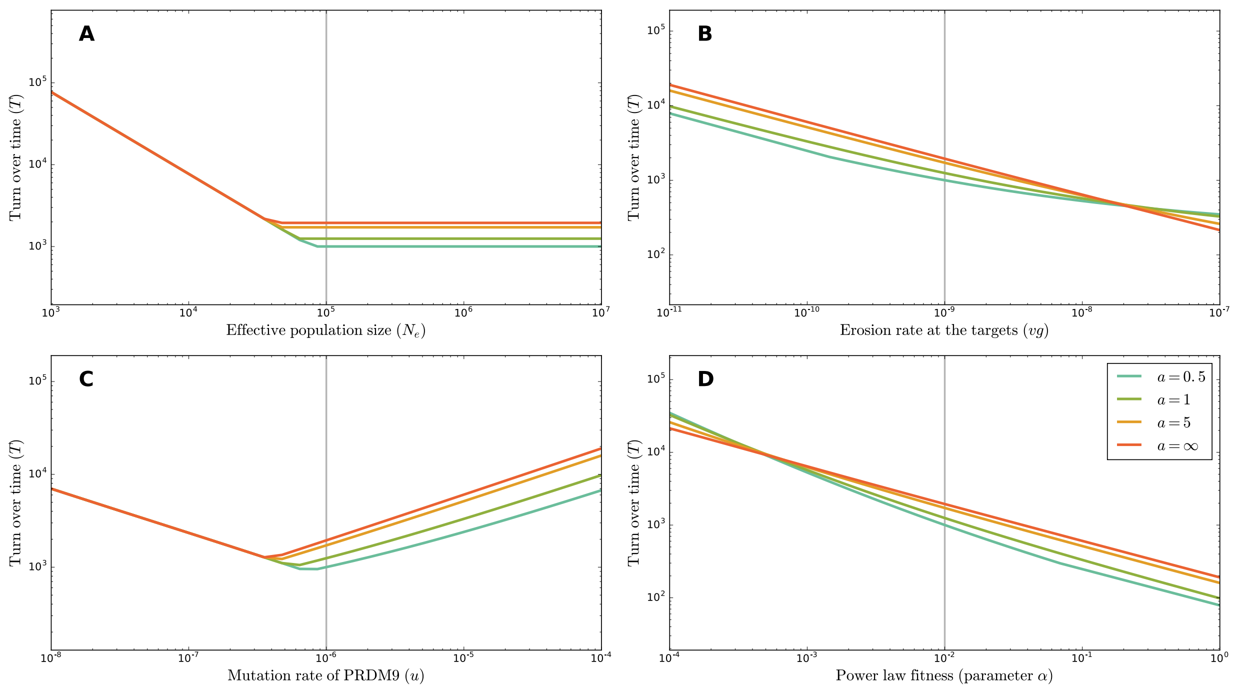

Figure S12. Turnover time T, calculated under the generalized mean-field approximation, as a function of effective population size N_e (A), erosion rate vg (B), mutation rate at the PRDM9 locus u (C) and the fitness parameter α (D) under the power-law fitness model and with variance in hot spot strength, with shape parameter $a = 0.5$, 1, 5 and $+\infty$.
